# Evaluating the Potential of Microdroplet Flow in Two-Phase Biocatalysis: A Systematic Study

**DOI:** 10.1021/acsami.4c15647

**Published:** 2025-01-10

**Authors:** Lanting Xiang, Jennifer Solarczek, Victor Krajka, Hui Liu, Lina Ahlborn, Anett Schallmey, Iordania Constantinou

**Affiliations:** † Institute of Microtechnology (IMT), Technische Universität Braunschweig, Alte Salzdahlumer Straße 203, DE-38124 Braunschweig, Germany; ‡ Center of Pharmaceutical Engineering (PVZ), Technische Universität Braunschweig, Franz-Liszt-Str. 35a, DE-38106 Braunschweig, Germany; § Institute for Biochemistry, Biotechnology and Bioinformatics, Technische Universität Braunschweig, Spielmannstr. 7, DE-38106 Braunschweig, Germany; ∥ Cognitive Systems Lab (CSL), University of Bremen, Enrique-Schmidt-Str. 5, DE-28359 Bremen, Germany

**Keywords:** Droplet microfluidics, Two-phase biocatalysis, Biphasic biocatalysis, Microdroplet flow, Slug flow

## Abstract

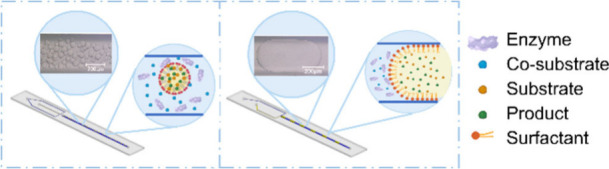

Two-phase biocatalysis in batch reactions often suffers from inefficient mass transfer, inconsistent reaction conditions, and enzyme inactivation issues. Microfluidics offer uniform and controlled environments ensuring better reproducibility and enable efficient, parallel processing of many small-scale reactions, making biocatalysis more scalable. In particular, the use of microfluidic droplets can increase the interfacial area between the two phases and can therefore also increase reaction rates. For these reasons, slug flow has been extensively used in two-phase biocatalysis in recent years. However, microdroplet flow has been largely neglected for this application despite its great potential. In this work, we performed biphasic biocatalysis in microfluidic droplets, both in microdroplets and slugs, as well as in batch, and systematically investigated the effect of various reaction parameters on the outcome of the reaction. We show that microdroplet flow outperforms the more commonly used batch and slug flow configurations for most reaction conditions by providing shorter substrate diffusion paths and larger interfacial area for the reaction. The potential trade-off between maximized mass transfer and possibly higher enzyme inactivation rates in small droplets with large surface-to-volume ratios was also investigated for the first time, and a pipeline was established to allow evaluation in other reactions. Finally, the effect of surfactant necessary for microdroplet stabilization was also investigated in all reaction setups for the first time, and it was shown that a properly selected surfactant can have a positive effect at low concentrations by creating more stable emulsions and smaller droplets, thus increasing the interfacial area between the two phases.

## 1 Introduction

The conversion of organic compounds using enzymes or other biocatalysts, such as whole cells, is known as biocatalysis. Biocatalysis has emerged as an important catalytic method in synthetic organic chemistry due to the many advantages of enzyme catalysts, especially their high chemo-, regio- and stereoselectivity enabling shorter and more simplified reaction routes.^1−3^ However, the effective application of enzymes, which commonly prefer aqueous reaction media, is often hampered by a low water solubility of the reaction substrates that are not hydrophilic in nature. This requires the concentration of such a substrate used in biocatalytic reactions to remain low (often well below the concentrations required for industrial applications), and/or the addition of a less polar cosolvent.^4^ Two-phase biocatalysis has emerged as an alternative solution to this problem by using two immiscible liquid phases, an aqueous and a nonaqueous phase; the latter commonly being an organic solvent^5,6^ or ionic liquid.^7^ In such two-phase setups, enzymatic reactions take place in the aqueous phase, into which the hydrophobic substrate diffuses at low concentrations close to the solubility limit. At the same time, the nonaqueous phase acts as a substrate reservoir and product sink. Thus, continuous product removal from the aqueous phase can be achieved, which shifts reaction equilibria and prevents possible substrate or product inhibition. Additional key advantages of two-phase biocatalysis include the use of exceptionally high substrate concentrations and the possibility for direct isolation of products through phase separation.^8^ Despite the many advantages of two-phase biocatalysis, there are also associated challenges that have kept this approach from wide application in industry and academia.^9^ One of these challenges is related to the low stability of many enzymes in the presence of nonaqueous media, resulting in reduced catalytic activity.^10^ Additionally, when performing liquid–liquid two-phase biocatalytic reactions in batch, mechanical stirring or shaking is required to ensure phase mixing and droplet formation, which can negatively affect enzyme stability and activity due to the shear forces. This approach also often leads to the formation of stable emulsions, complicating phase separation and product collection.^11^ Achieving uniformly sized droplets in batch systems is also challenging, as stirring and shaking introduce variable shear forces unpredictable droplet coalescence or breakup, and difficulty in precisely controlling agitation intensity, all of which contribute to inconsistent droplet sizes.^12^ These challenges can be overcome with the use of microfluidic bioreactors as a replacement for traditional batch reactions. Finally, microfluidics enable continuous flow reactions and offer higher surface-to-volume ratios, shorter diffusion paths, as well as greatly increased mass and heat transfer rates.^13^ When applied to two-phase biocatalysis, these characteristics could greatly contribute to overcoming the aforementioned limitations of batch reactions by promoting controlled contact between the aqueous and nonaqueous phases.

Utilizing laminar flow in microfluidics, biphasic biocatalysis can be achieved on chip when the aqueous and nonaqueous phases are introduced into the microfluidic device side by side, offering an interface through the length of the channel across which diffusion can occur.^14^ Alternatively, microfluidic droplets can also be utilized. In this case, enzyme-catalyzed reactions can take place at the interface between the microfluidic droplet and the continuous phase that carries the droplets. Although microfluidic droplets are often more difficult to produce compared to laminar flow, they offer larger surface area-to-volume ratios and shorter diffusion paths that result in faster mass transfer and reduced reaction time.^15^ Additionally, microfluidic systems allow for uniform droplet formation under gentler conditions, thus preserving enzyme integrity and activity by avoiding large shear forces.^16^ Finally, the precise control over droplet size in microfluidics allows for controlled and quantifiable mass transfer and surface area optimization, for improved reaction efficiency.^17^

**Figure 1:**
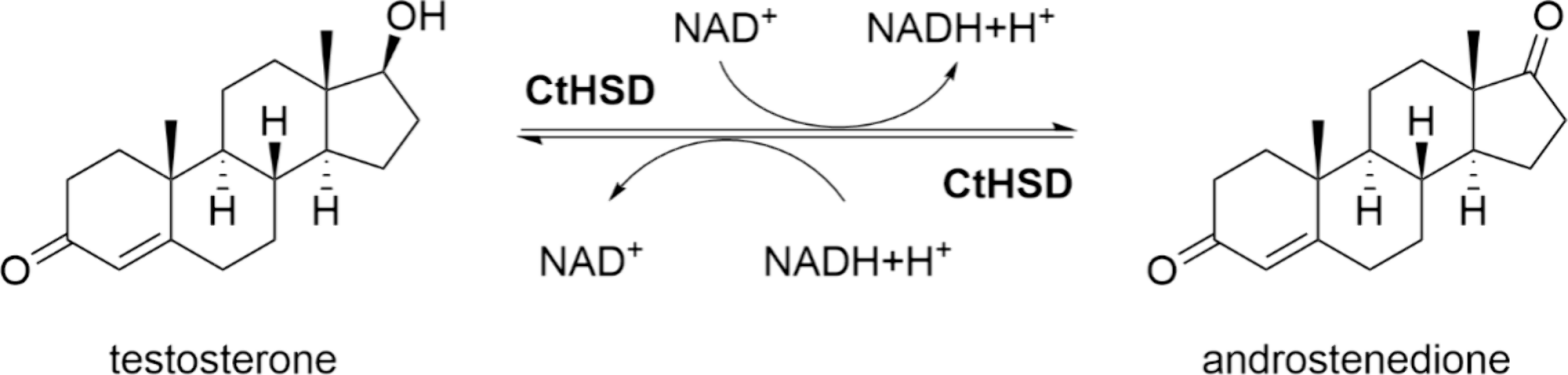
Reaction scheme of the reversible conversion of testosterone to androstenedione using the hydroxysteroid dehydrogenase from *C. testosteroni* (CtHSD).^22^

To evaluate the suitability of microfluidic droplets for two phase biocatalysis, we studied the reversible *Nicotinamide adenine dinucleotide* (NAD)-dependent conversion of testosterone to androstenedione using the 3β/17β-hydroxysteroid dehydrogenase from *Comamonas testosteroni* (CtHSD) as model reaction (Figure 1).^18^ More than 300 different steroid-based *active pharmaceutical ingredients* (APIs) are approved today, that are used, e.g., as contraceptives, anti-inflammatories or immunosuppressives.^19^ The production of such steroidal drugs in the pharmaceutical industry often involves biocatalytic reaction steps, e.g., catalyzed by cytochrome P450 monooxygenases^20^ or hydroxysteroid dehydrogenases,^21^ among others, despite the rather low solubility of steroids in aqueous reaction media. Thus, two-phase biocatalysis could be a valuable option in these cases.

In this work, we compare biphasic biocatalysis in microdroplet flow, slug flow and in batch. The effect of microdroplet size/geometry on the outcome of two-phase biocatalytic reactions was evaluated using *space-time yield* (STY) as our main metric. A comparison with reactions performed in batch configurations was carried out in order to decipher the potential positive or negative effects of microfluidic droplet-based two-phase biocatalysis on-chip. For the microfluidic experiments, we designed, fabricated, and tested glass microfluidic devices. We found that both microfluidic approaches outperformed batch reactions in terms of STY. The highest STY was reached in reactions performed in microdroplets, demonstrating the potential of this technology in comparison to the commonly used slug flow. As the main difference between the two microfluidic droplet systems is droplet size and thus the interfacial area between the two phases, we rigorously studied the effect of varying interfacial area on STY. We show that in microdroplet flow, STY is proportional to the interfacial area between the two phases and inversely proportional to the microdroplet diameter. Additionally, since surfactants are necessary for microdroplet stability, the effect of surfactant concentration on STY was also investigated for the first time.

## 2 Materials and Methods

### 2.1 Reaction Description

The *3β/17β-hydroxysteroid dehydrogenase* (17β-HSDs) used in this work was derived from *C. testosteroni* and is naturally involved in the degradation of testosterone and other steroid compounds by catalyzing the reversible reduction/dehydrogenation of oxo/hydroxy groups at positions 3 and 17 of the steroid backbone at the expense of NADH/NAD^+^ as cofactor. This enzyme is well-characterized in literature^23,24^ and has been selected due to its high activity^25^ as well as easy heterologous production in *Escherichia coli* (see Supporting Information (SI) for experimental details).

**Figure 2:**
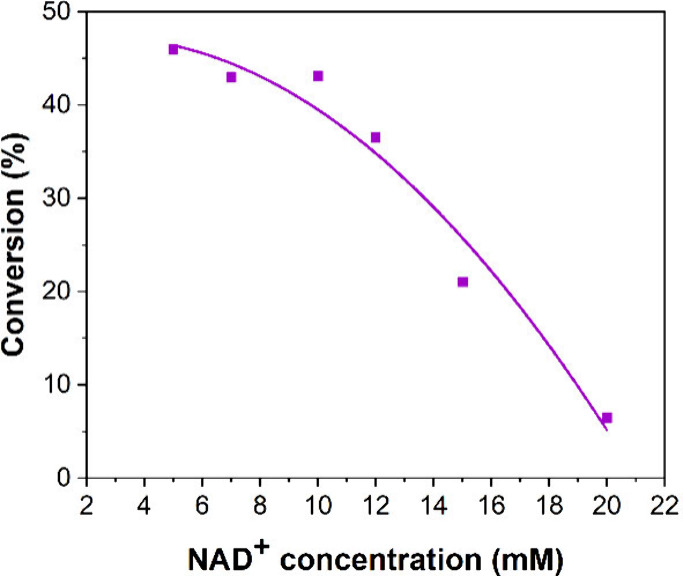
Inhibition of CtHSD activity by increasing NAD^+^ concentration. Conversion of testosterone was determined after 2 min at 25 °C and 900 rpm shaking from reactions containing 20 mM Tris-HCl buffer, 25 mM NaCl, pH 9, 50 μg/mL CtHSD, 2.5 mM testosterone, 10% TBME and 5–20 mM NAD^+^. Reaction workup and high-performance liquid chromatography (HPLC) analysis were performed as described in Section 2.3 and SI.

The dehydrogenation of testosterone to androstenedione is a reversible reaction (Figure 1) with an equilibrium constant of approximately 2.2 (Figure S1, see also SI for experimental details). Thus, to push the equilibrium to the side of the product androstenedione, the NAD^+^ concentration should be used in excess. Interestingly, however, our studies revealed that CtHSD activity is inhibited at increasing NAD^+^ concentration, resulting in a significant decrease in conversion at NAD^+^ concentrations above 10 mM (Figure 2). Thus, we generally limited NAD^+^ concentrations to ≤10 mM. Increasing the concentration of enzyme generally accelerates the biocatalytic reaction rate and results in shorter reaction times to reach the equilibrium but, at the same time, the enzyme amount significantly contributes to the costs of the reaction. In this work, a 50 μg/mL enzyme concentration was used as it remains financially reasonable, while allowing high conversion rates at short reaction times (see Figure S2).

To perform two-phase reactions, the solubility of testosterone in different apolar solvents such as *n*-heptane, *tert-butyl-methyl-ether* (TBME), *diisopropyl ether* (DiPE), cyclohexane and *n*-octane was studied. Testosterone concentrations in the organic phase higher than 10 mM could be reached only when using DiPE and TBME as solvents (12 mM in DiPE and 50 mM in TBME). Similar solubilities in DiPE (10 mM) and TBME (50 mM) were also obtained for the product androstenedione. At the same time, half-lives of CtHSD (as a measure of enzyme stability) in batch reactions using 10% DiPE or TBME were determined at 8.1 or 2.4 min, respectively (Figure S3; see SI for experimental details). CtHSD appears slightly more stable in DiPE than TBME, however, TBME allows the use of significantly higher testosterone concentrations in the organic phase and thus also in the reaction and was therefore chosen as the apolar solvent for the following investigations.

### 2.2 Reactions in Batch and in Microfluidics

**Figure 3:**
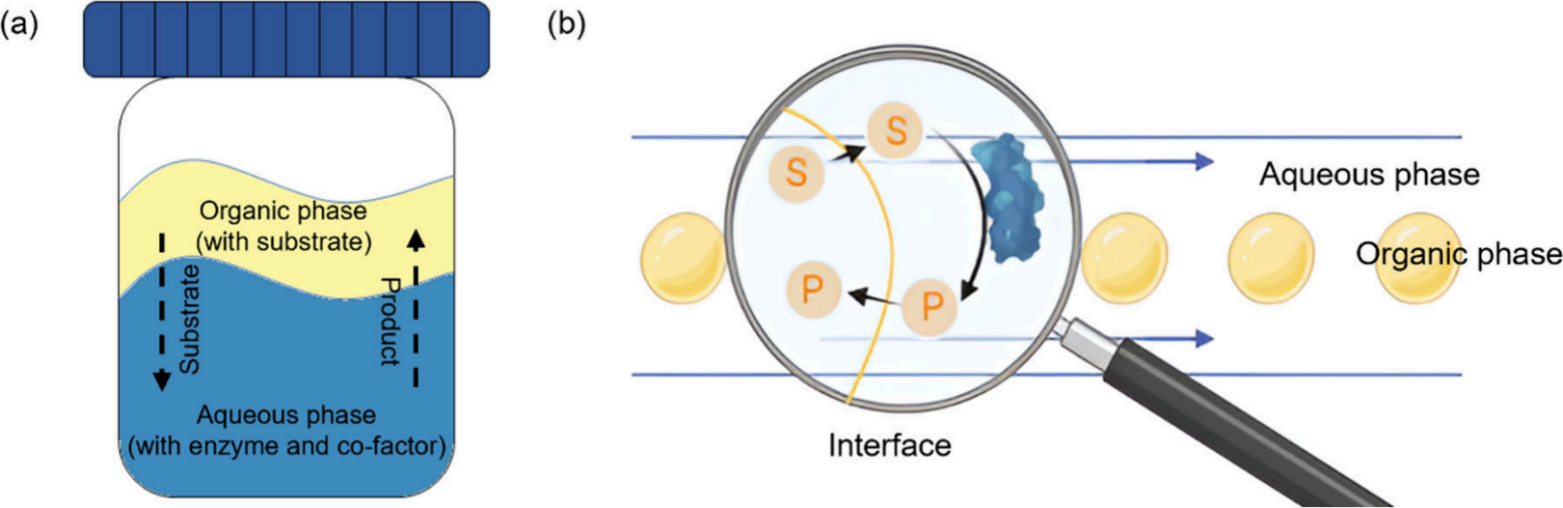
(a) Conventional two-phase biocatalysis setup, the yellow area represents the organic phase and the blue area the aqueous phase. The organic phase contains the substrate. The enzyme and cofactor reside in the aqueous phase. (b) Two-phase biocatalysis in droplet microfluidics. Microdroplets containing the organic phase surrounded by the aqueous phase travel along the microchannel. A magnified view of the organic/aqueous interface shows the substrate (S) diffusing to the liquid/liquid interface. The formed product (P) is collected via the organic phase.

Traditionally, biphasic biocatalytic reactions are performed in batch. In this work, we compare enzyme-catalyzed, biphasic testosterone dehydrogenation performed in batch and in droplet microfluidics. *Space-time yield* (STY) was chosen as our main metric as it accounts for differences in reaction time and reaction volume, essential when comparing reactions performed in batch to reactions performed in microfluidics. STY describes the amount of product (in mol) that can be produced per unit time (h) and unit volume (cm^3^) and it is commonly used to evaluate biocatalytic processes. As mentioned before, TBME was chosen as the organic phase for our experiments based on the solubility of the substrate testosterone. The enzyme, CtHSD, and cofactor NAD^+^ were supplied in buffered aqueous media (20 mM Tris-HCl, 25 mM NaCl, referred to as the aqueous phase for this reaction) with a pH of 9, the optimal pH of CtHSD. A general schematic of a biphasic biocatalytic reaction performed in batch is shown in Figure 3a. In general, biphasic biocatalysis is driven by diffusion. In batch, the organic phase containing the substrate and the aqueous phase containing the enzyme (and in this case also the cofactor) are mixed by shaking or stirring in order to increase the contact surface area between the two phases and facilitate mass transfer. When a hydrophobic substrate such as testosterone is to be converted, the biocatalytic reaction is expected to take place close to the liquid–liquid interface with only little substrate diffusing further into the aqueous phase. The resulting reaction product, here androstenedione, diffuses back and also accumulates in the organic phase, where it can be collected for subsequent analysis and product isolation. Although batch reactions are standard in two-phase biocatalysis, they do not come without limitations. For example, vigorous shaking or stirring can cause enzyme denaturation. At the same time, the formation of stable emulsions during mixing impedes phase separation for product collection. Additionally, the amount and type of the organic phase used can also have a negative effect on the reaction, as it can inhibit the enzyme, affect its molecular structure, and influence enzyme selectivity.^26^

Figure 3b shows a two-phase biocatalytic reaction carried out in droplet microfluidics. In this experimental setup, microdroplets (i.e., the dispersed phase) contain the organic phase. Similar to batch configurations, the aqueous phase (here the continuous phase) contains the enzyme and cofactor. This microfluidic droplet configuration is known as oil-in-water (O/W) configuration. Similar to batch reactions, the biotransformation of testosterone in microfluidic droplets takes place close to the liquid–liquid interface and the formed product is collected via the organic phase. Since microfluidic droplets are only a few tens to few hundreds of micrometers in diameter/length, diffusion paths for substrate within the droplet are very short and thus diffusion times are also very short (calculated to be less than 1s, see Section 3.4). That, in addition to the increased interfacial area is expected to result in a much faster reaction rate, i.e., the rate of product formation is expected to be higher compared to batch reactions. On the other hand, the large interfacial area could lead to larger enzyme inactivation similar to what is observed in emulsions formed in batch. The trade-off between increased mass transfer vs large enzyme inactivation at larger interfacial areas will be extensively investigated in the following sections. Finally, in microfluidic systems, droplet separation and collection, for example via a separate channel, can be integrated on chip, providing a streamlined process that requires no emulsion separation for product collection outside of the device. On chip separation of the dispersed and continuous phases can be achieved through several techniques, including wettability-based phase separation,^27^ pressure-regulated outlet separation,^28^ and electric field-driven methods such as electrophoresis and dielectrophoresis.^29^ While very few examples are found in the literature for biphasic biocatalysis performed in spherical microdroplets,^30,31^ the larger surface area-to-volume ratio inherent to smaller droplets, along with the shorter diffusion paths for substrate and product could benefit biocatalytic efficiency. To date, the majority of published work utilizing droplets for biphasic biocatalysis is based on slug flow.^32−38^ Details about the microfluidic device layouts and dimensions that used in this work, and droplet formation mechanisms are provided in SI, along with Figure S4.

### 2.3 Experimental Setup for Two-Phase Biocatalysis in Batch and in Microfluidics

The complete experimental procedure, including the experimental setup and conditions for two-phase biocatalysis in batch and in microfluidic droplets is discussed in detail in the SI. As the effect of several parameters on the outcome of the reaction was investigated, experimental details related to surfactant concentration (Tween 20), organic phase ratio, and substrate concentration are presented. Further information related to the experimental setups (for example, microfluidic device fabrication, microdroplet formation in microfluidics), sample preparation, and analysis steps can also be found in SI along with Figure S5 and S6.

### 2.4 Chemicals

All chemicals used in this study were of analytical grade or higher and used without prior purification. Testosterone and Tween 20 was purchased from Sigma-Aldrich (Steinheim, Germany), NAD^+^ was obtained from Prozomix Limited (Haltwhistle, United Kingdom). All other chemicals were purchased from Sigma-Aldrich (Steinheim, Germany), Fisher Scientific (Schwerte, Germany), Carl Roth (Karlsruhe, Germany) or VWR (Darmstadt, Germany).

### 2.5 Microfluidic Droplet Detection for Interfacial Area Calculations

One of the parameters that determines the outcome of two-phase biocatalysis is the total interfacial area between the organic and aqueous phases. Larger interfacial area could provide more space for the reaction to take place, but at the same time, more contact between the organic phase and the enzyme could lead to enzyme inactivation. In order to evaluate and compare two-phase biocatalytic reactions performed in microdroplets and in slugs, it is important to evaluate the total available surface area in each system. Since in this work microfluidic devices were fabricated in glass, a digital microscope (Keyence, model VHX-5000, equipped with a 1/1.8-in., CMOS image sensor) was used to image microfluidic droplets produced under different experimental conditions. To determine the size of microdroplets, images we taken using the following settings: 100× magnification, 1600 × 1200 pixels, 0° camera tilt, 50 frames/second, mixed illumination (full coaxial and ring illumination), and saved as Tiff files. An unbiased method was then established that allowed the calculation of the number and the diameter of microfluidic droplets from the acquired microscopy images. The details of the pipeline developed for microfluidic droplet detection and interfacial area calculations can be found in SI, along with Figures S7, S8 and Table S1.

## 3 Results and Discussion

To evaluate the suitability and potential advantages of microfluidic droplets for use in two-phase biocatalysis, we performed testosterone conversion to androstenedione by CtHSD in batch as well as in microdroplet and slug flows and compared reaction outcomes in terms of space-time yield (STY). For this, a parameter study was conducted on the influence of enzyme concentration, NAD^+^ concentration, substrate concentration, solvent ratio and reaction time in each reaction configuration. In addition to these parameters, the flow rate of each phase and the surfactant concentration were optimized for microfluidic reactions, as these parameters affect microfluidic droplet size and can therefore greatly affect the outcome of the reaction. Additionally, in microfluidic reactions, experimental parameters are often interrelated. For example, the organic-to-aqueous flow rate ratio not only determines the ratio between the organic and aqueous phases but can also affect the droplet size and thus the interfacial area available for the reaction. Therefore, reaction parameter optimization in microfluidic droplets is not a straightforward process. Below, we describe the process of optimizing STY in microfluidic reactions, compare it to reactions performed in batch and discuss the effect of experimental parameters on the reaction outcome.

### 3.1 The Effect of Surfactants on the Reaction

**Figure 4:**
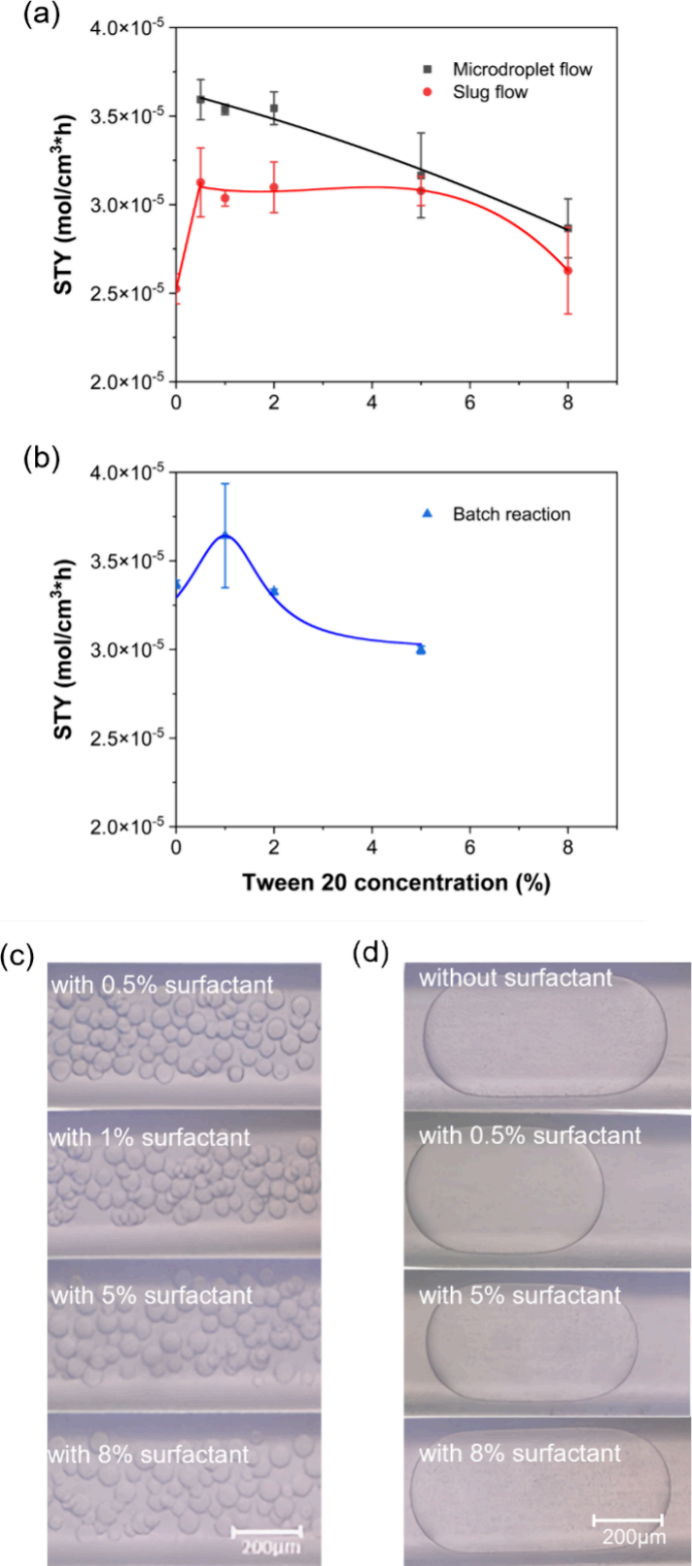
a) STY in microdroplet flow and slug flow for increasing surfactant concentrations. Standard deviation is shown in error bars based on two experimental replicates. In both microdroplet flow and slug flow, STY peaks at 0.5% Tween 20 concentration. Experiments were performed under the same reaction conditions (enzyme concentration 50 μg/mL, NAD^+^ concentration 5 mM, substrate concentration 2.5 mM, 25% organic phase in microdroplet flow and slug flow and 2 min reaction time). b) STY in batch reactions for increasing surfactant concentrations. Experiments were performed under the following reaction conditions: enzyme concentration 50 μg/mL, NAD^+^ concentration 5 mM, substrate concentration 2.5 mM, 10% organic phase in batch reaction and 2 min reaction time. Digital microscope images of c) microdroplet flow at 0.5%, 1%, 5%, 8% and d) slug flow at 0, 0.5%, 5%, and 8% surfactant concentrations. When Tween 20 was added to the aqueous phase, shorter slugs were formed when experimental conditions remained otherwise unchanged. As Tween 20 concentration increased, the slug length also increased. In microdroplet flow shown in c), two distinct microdroplet sizes can be seen as a result of Marangoni flow patterns arising from the use of surfactant. No change in microdroplet size was observed with increasing concentrations of Tween 20. Experiments were performed at 6 μL/min aqueous phase flow rate and 2 μL/min organic phase flow rate.

Surfactants are amphiphilic molecules that have a hydrophilic end and a hydrophobic end. Due to their amphiphilic nature, they adsorb at interfaces and help stabilize them, in our case, the interface between the organic and aqueous phases. To establish a stable and reproducible microdroplet flow, surfactants need to be used, as without them, microdroplets fuse with each other and lower their surface energy by forming larger droplets. In addition to stabilization, the surfactant concentration can be used to control the size of microdroplets in two-phase liquid flow systems.^39,40^ In this work, the nonionic surfactant polysorbate 20 (Tween 20) was added to the aqueous phase to promote stable microdroplet generation at concentrations ranging from 0 to 8%,% (v/v). Tween 20 was chosen because it is chemically stable and can be considered nontoxic, which makes it a very popular choice for applications in pharmaceutical and food production.^41^ The *critical micelle concentration* (CMC) of Tween 20 is approximately 40 μM.^42^ In this study, the minimum concentration of Tween 20 used in microfluidic systems was 0.5% (v/v), which is significantly above its CMC. This high concentration ensures effective droplet stabilization and prevents coalescence, thereby enhancing the consistency of the microdroplet system. We performed microfluidic reactions using the following parameters: 50 μg/mL enzyme concentration, 5 mM NAD^+^ concentration, 2.5 mM substrate concentration, 25% organic phase and 2 min reaction time. These experimental conditions were selected as they resulted in the highest STY for microfluidic reactions (see Section 3.2 below). In microdroplet flow, the minimum concentration of surfactant that leads to stable droplets at low flow rates is 0.5%. When no surfactant was added to the aqueous phase (i.e., at 0% Tween 20), stable microdroplets could not be formed, and therefore no data could be recorded. While some surfactants have been shown to negatively affect the catalytic activity of enzymes,^43^ a high STY was achieved when a low concentration of Tween 20 (0.5%) was added to allow the formation of microdroplets (Figure 4a). Although stable slugs can be formed without surfactants, the impact of Tween 20 on slug formation and reaction efficiency was also investigated. Surprisingly, STY also increased when low concentrations of Tween 20 were used in slug flow (Figure 4a). At concentrations of Tween 20 over 5%, STY dropped but remained above the STY calculated for slug reactions performed without the use of surfactant. We believe that this increase in STY is caused by an initial reduction in slug droplet length and a corresponding increase in the total interfacial area available for the reaction (Figure 4d). When droplets started to get larger again at Tween 20 concentrations above 5%, STY drops as shown in Figure 4a and discussed in more detail below. The observed change in slug length is due to changes in the interfacial tension between the dispersed and continuous phases when surfactant is added to the aqueous phase. At low surfactant concentrations, we expect that the interfacial tension would be reduced as previously reported, which facilitates the breakup of the dispersed phase into smaller droplets.^44^ The increase in slug size observed at surfactant concentrations over 5% may be due to several contributing factors, one of them being steep surfactant concentration gradients inducing Marangoni stresses. Such gradients can be found near the flow-focusing region where surfactants can accumulate, as in this region, the adsorption of surfactant molecules onto the droplet interface may not keep pace with the droplet generation rate, particularly under rapid production conditions, leading to a buildup of surfactant in the surrounding fluid. Another reason could be an increase in viscosity at higher Tween 20 concentrations, which increases flow resistance and slows down the pinch-off process, which in turn allows the dispersed phase to form larger droplets, even when interfacial tension remains relatively constant.^45^ We have indeed measured the viscosity of the aqueous phase at 0% Tween 20 and at 8% Tween 20 and have found that it increases from 1.0078 ± 0.0041 mm^2^/s to 1.6586 ± 0.0058 mm^2^/s, corroborating our hypothesis (see SI for experimental details).

Other contributing factors might include Marangoni effects arising from local concentration gradients at the slug interface, which generate surface tension differences along the interface.^46^ These gradients create Marangoni flows that can stabilize the interface, resisting deformation or breakup. Additionally, more complex phenomena such as nonequilibrium adsorption could also play a role, although we currently have no experimental evidence to support this. In this work, all STY data were derived from at least three replicated experiments.

Smaller droplets allow for a faster diffusion of substrate due to shorter diffusion lengths and offer more interfacial area for mass transport and the reaction, all of which boost STY. As shown in Figure 4a, higher concentrations of Tween 20 (in the case of slug flow concentrations above 5%) result in a decrease in STY for all three investigated systems. In contrast to slug flow, we have not observed any significant change in the average size of microdroplets in the range of 0.5–8% (Figure 4c). At the low flow rates used in our experiments (aqueous phase 6 μL/min and organic phase 2 μL/min), however, we observed the formation of microdroplets of two distinct diameters (76 ± 2.4 and 51 ± 2.4 μm). We attribute this bimodal size distribution to the fact that our system likely operates in a jetting or transitional regime, as revealed by a closer examination of the junction (Figure S9, supplemental movie). In the jetting regime, smaller satellite droplets form because the thinning liquid thread between two primary droplets can break into smaller fragments before fully retracting. In the presence of surfactant, and especially in the case of nonuniform surfactant distribution, surface tension gradients are created along the jet, which induce Marangoni flows, which can either stabilize or destabilize parts of the jet, influencing how and where it breaks.^47^ A nonuniform surfactant distribution is reasonable to expect since, as discussed above, during droplet formation, surfactants do not distribute evenly across the interface of each droplet primarily due to local concentration differences and adsorption kinetics. Despite the occurrence of bimodal droplet sizes, the overall size distribution remained consistent across all experimental conditions. Thus, we hypothesize that the drop in STY in microdroplet flow observed at higher Tween 20 concentrations is mainly due to hindered mass transport across the microdroplet interface that is lined with surfactant molecules. When the concentration of surfactant increases beyond a certain level, the intermolecular gap between the surfactant molecules arrayed at the interface between the organic phase and the aqueous phase (i.e., the periphery of the droplet) decreases as more surfactant molecules crowd the interface and act as a barrier.^48^ A direct negative impact of Tween 20 on CtHSD catalytic activity could be ruled out, as we did not observe a significant change in enzyme activity when measured in a monophasic system in the presence of Tween 20 (Figure S10).

In batch reactions, the addition of surfactants can also help to form more stable emulsions, thus increasing the interfacial area between the two phases. Similar to microfluidic reactions, when low concentrations (1%) of Tween 20 were added to the aqueous phase of reactions performed in batch, STY also increased. In batch reactions, 10% organic phase ratio was used (as opposed to 25% used in microfluidic experiments) as it results in the highest STY (see Section 3.2 below). At 1% Tween 20, STY was ∼8% higher compared to reactions performed without surfactant (Figure 4b). When the concentration of Tween 20 increased to 5%, STY dropped by ∼10%. This demonstrates that low surfactant concentrations are also beneficial for reactions performed outside of microfluidic systems, especially ones that rely on mass transfer in emulsions. Despite the fact, to align with standard batch practices and enable a direct comparison between conventional batch reactions and microfluidic-based reactions, no surfactant was added to the batch reactions discussed in the following sections.

### 3.2 The Effect of Aqueous-to-Organic Phase Ratio on the Reaction

**Figure 5:**
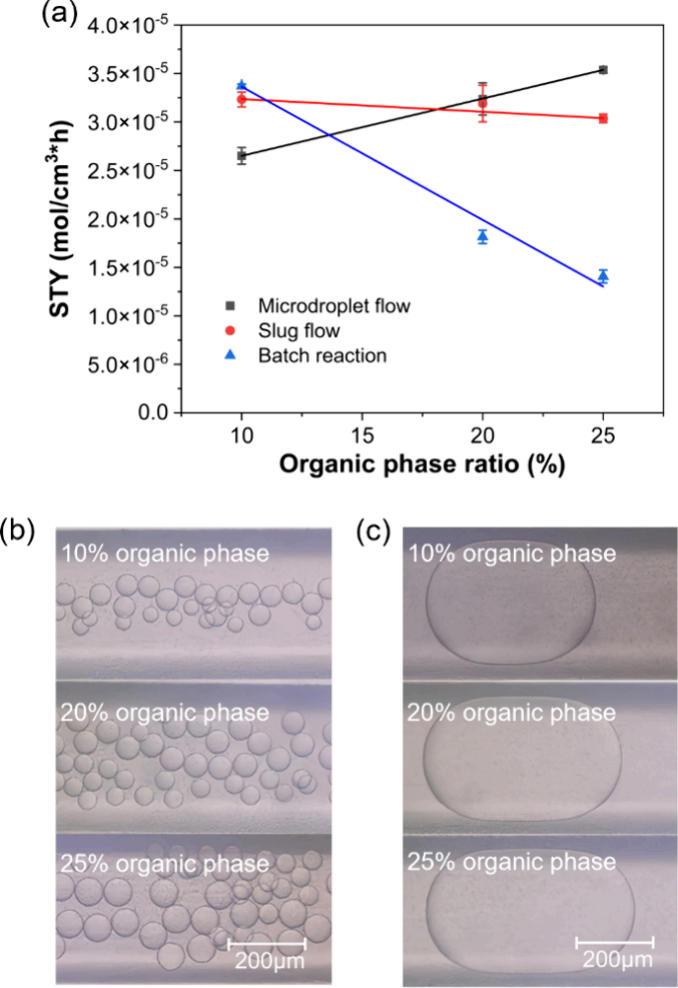
(a) STY in batch reactions, microdroplet flow, and slug flow at 10%, 20% and 25% organic phase ratio (total flow rate 8 μL/min). In batch (blue triangles), as the organic phase proportion increased the overall STY decreased likely due to enzyme inactivation. In microdroplet flow (black squares), the STY increased at increasing organic phase proportions. In slug flow (red circles), a slight decrease in STY was observed at higher organic phase proportions. (b) Digital microscope images of microdroplets generated at 10%, 20% and 25% organic phase ratios. As the organic phase proportion increased, the average diameter of generated microdroplets also increased along with microdroplet population and total calculated interfacial area. (c) Digital microscope images of slugs generated at 10%, 20% and 25% organic phase ratios. An increase in slug length was observed at higher organic phase proportion, which translates into longer distances the substrate needs to travel to reach the liquid–liquid interface where it can be converted, explaining the decrease in STY. In slug flow, the total calculated interfacial area remained largely unchanged. Experiments were performed under the same reaction conditions (enzyme concentration 50 μg/mL, NAD^+^ concentration 5 mM, substrate concentration 2.5 mM, 1% surfactant (only in microflow), and 2 min reaction time).

In two-phase biocatalysis, increasing the amount of organic phase would enable us to use more substrate in the reaction but would not necessarily yield more product, as organic solvents are known to inhibit or inactivate the enzymes used as catalysts.^10^ The effect of the organic phase proportion on STY in CtHSD-catalyzed testosterone dehydrogenation is shown in Figure 5a for reactions performed in microdroplets, microfluidic slugs, and in batch. In batch reactions, an organic phase ratio over 10% leads to decreasing STY. Given that other experimental parameters such as enzyme concentration, substrate concentration and reaction time remained constant, we believe that the lower STY results from enzyme inactivation at higher organic phase proportions. The problem of enzyme inactivation at an increasing organic phase proportion can be mitigated when droplet microfluidics are used due to organic phase compartmentalization. The black line in Figure 5a shows the STY of reactions performed in microdroplet flow for different organic phase proportions, adjusted by changing the flow rate ratio between the two phases. In complete contrast to the outcome of batch reactions, the STY greatly increases with increasing organic phase ratio up to a proportion of 25% (higher proportions also discussed in Section 3.4). These results are consistent with the phenomenon known as spontaneous reaction acceleration in microdroplets, that refers to the unexpected and often significant increase in reaction rates for enzymatic (and other catalytic) reactions that occur in microdroplets, compared to traditional bulk solutions.^49^ To understand the origin of this effect, changes in droplet population and geometry with increasing organic phase ratios were investigated using microscopy (Figure 5b and 5c). It was found that at higher organic phase proportion, the population of microdroplets was larger: 95 droplets/*mm*^2^ at 10% to 122 droplets/*mm*^2^ at 25%. The diameter of microdroplets also increased at higher organic phase proportion from 56 ± 1.3 and 36 ± 1.6 μm (bimodal) at 10% to 76 ± 2.4 and 51 ± 2.4 μm at 25%. In microdroplet flow systems, the total interfacial area within one microscope region of interest (ROI) at 100× magnification (∼2340 μm width ×400 μm length) is the sum of the surface areas of all microdroplets, which can be calculated from the average diameter and population of microdroplets. Despite the increase in microdroplet diameter, the calculated total interfacial area (see Section 2.5 and SI) between the two phases more than doubled from (6.4 ± 0.09) × 10^5^ μm^2^ at 10% to (1.5 ± 0.02) × 10^6^ μm^2^ at 25% organic phase proportion. This indicates that in microdroplet reactions, the positive effect of an increased interfacial area at higher organic phase proportions outweighs the negative effects of the organic phase on enzyme activity. Indeed, we have measured a residual enzyme activity of around 100% after microfluidic reactions (Table S2).

In slug flow, a slight decrease in STY was observed at a higher organic phase proportion (Figure 5a, red line). This is due to an increase in slug length, that in turn increases the diffusion length for the substrate (i.e., the distance the substrate needs to travel before it is converted) while the interfacial area remained largely unchanged at (2.0 ± 0.08) × 10^5^ μm^2^ as it is primarily located at the ends of slugs, where the radius of curvature was constant. This of course requires that the total number of slugs inside the device also remained largely constant, which is reasonable since slugs become longer and the spacing between them becomes shorter due to higher organic phase proportion.

We have so far established that microfluidic systems can significantly improve the productivity of two-phase biocatalytic reactions compared to traditional batch configurations by allowing the use of higher organic phase proportions without the risk of enzyme inactivation. When comparing microdroplet flow to slug flow, we found that slug flow has an advantage at low organic phase proportions, while microdroplet flow results in higher STY at high organic phase proportions. At 25% organic phase ratio, the total interfacial area of microdroplet flow is about five times higher than that of slug flow. The larger interfacial area and shorter diffusion paths inherent to smaller droplets become the main drivers of reaction rate, resulting in higher STY for microdroplet flow compared to slug flow.

### 3.3 The Effect of Substrate Concentration on the Reaction

**Figure 6:**
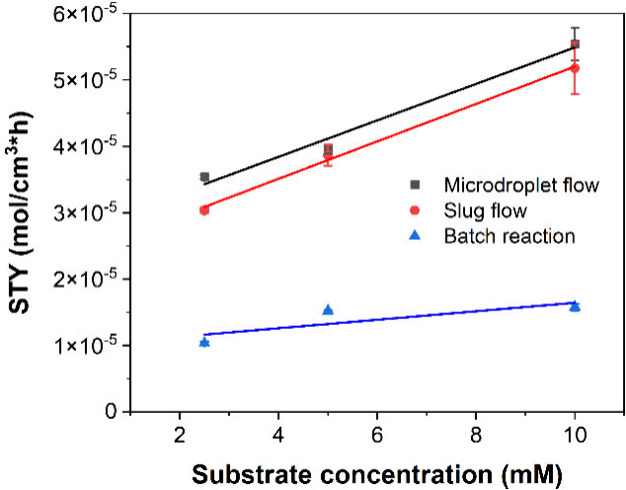
STY at increasing substrate concentrations for batch, microdroplet, and slug flow reactions. As substrate concentration increased, STY increased steeply for reactions performed in microdroplet flow and slug flow. In contrast, the change observed in STY for reactions performed in batch was minimal. This is due to higher enzyme inactivation in batch reactions. Experiments were performed using the following reaction conditions: enzyme concentration 50 μg/mL, 5 mM NAD^+^ concentration at 2.5 mM substrate concentration, 10 mM NAD^+^ concentration at 5 mM and 10 mM substrate concentration, 1% surfactant (only added to microflow reactions), 2 min reaction time for microfluidic reactions and 10 min for batch reactions. In microfluidic experiments a 25% organic phase ratio was used for all substrate concentrations. In batch reactions, 10% organic phase ratio was used for 2.5 and 5 mM substrate concentration, while 20% organic phase ratio had to be used for 10 mM substrate concentration.

As high substrate concentrations are always desired for biocatalytic application, we also studied the effect of substrate concentration on STY in batch and microflow. Since the solubility of testosterone in TBME is limited to 50 mM, we only investigated testosterone concentrations up to 10 mM in the final reaction volume. As shown in Figure 6, STY increases in batch reactions and reactions performed in microfluidic droplets at an increasing substrate concentration. Specifically, STY steeply increases with increasing substrate concentrations in reactions performed in microfluidic droplets, from (3.54 ± 0.029) × 10^–5^ to (5.54 ± 0.025) × 10^–5^ for microdroplet flow and from (3.04 ± 0.049) × 10^–5^ to (5.18 ± 0.039) × 10^–5^ for slug flow. This is because the increase in testosterone concentration in the organic phase causes a higher mass transfer across the liquid–liquid interface and, thus, yields a higher testosterone concentration in the aqueous phase. Therefore, more substrate is available for conversion by the enzyme resulting in higher STY. We roughly determined the partition coefficient of testosterone in our two-phase system composed of 20 mM Tris-HCl, 25 mM NaCl pH 9.0 as the aqueous phase and TBME as the organic phase in batch (see SI for details, Table S3). This indicated that only approximately 0.5% of the testosterone supplied in the organic phase actually ends up in the aqueous phase. In case of a 2.5 mM substrate concentration in the reaction (i.e., 10 mM testosterone in the organic phase when 25% TBME are used), this means that only 50 μM testosterone are present in the aqueous phase and this value increases to roughly 200 μM in a reaction with 10 mM substrate concentration. Considering that according to literature CtHSD exhibits a K_M_ (Michaelis constant, indicates the enzyme’s affinity for the substrate) value of 11.8 μM and a *k*_cat_ (Turnover number, represents the enzyme’s catalytic efficiency under saturating substrate conditions) of 200 s^–1^ for testosterone dehydrogenation in 20 mM Tris-HCl buffer at pH 8.5,^50^ our measured testosterone concentrations in the aqueous phase are indeed still close to the K_M_ of the enzyme and will thus impact catalytic efficiency. Moreover, since this partition coefficient was determined in batch with mixing of both phases via shaking for 1 h, it can be assumed that the actual testosterone concentration in the aqueous phase in microflow is even lower and will further depend on the available interfacial area (*vide infra*).^52^ In contrast, in our reactions performed in batch the increase in STY was much smaller, from (1.03 ± 0.023) × 10^–5^ at 2.5 mM substrate concentration to (1.58 ± 0.050) × 10^–5^ at 10 mM testosterone concentration. In this system, the organic phase proportion had to be increased from 10% (used in reactions with 2.5 and 5 mM testosterone) to 20% to achieve the intended 10 mM substrate concentration. Thus, the smaller increase in STY in batch reactions is probably due to a higher enzyme inactivation rate at 20% organic phase ratio (compare Figure 5a), which curbs the positive effect of increasing substrate concentration. For reactions performed in microfluidic droplets instead, a constant organic phase proportion of 25% was used for all substrate concentrations.

### 3.4 The Effect of Droplet Size and Interfacial Area on Microdroplet Flow Reactions

The experimental results discussed in the sections above demonstrate the great potential of microdroplets in two-phase biocatalysis. As such reaction systems are hugely underutilized in research and development, a deeper understanding of how microdroplet properties can affect the outcome of a reaction is still lacking. We specifically looked at the effect of droplet size and overall available interfacial area on STY in microdroplet flow systems. When flow-focusing microfluidic devices are used to produce microdroplets for two-phase biocatalysis, three parameters need to be taken into account in order to reproducibly control the microdroplet size and number: the concentration of surfactant, the aqueous-to-organic phase ratio and the flow rates used.^51−53^ For example, as shown in Figure 5b when the proportion of organic phase in the overall solution is increased, droplets become larger and more numerous. The number of microdroplets also increases when flow rates increase; however, the size of microdroplets decreases. To evaluate the effect of microdroplet size on STY, we varied the microdroplet diameters by 1) controlling the flow rate of the system without changing the aqueous-to-organic phase ratio, and 2) increasing the organic phase proportion (Table 1). Droplet diameters were measured for four experimental conditions, the total interfacial area was determined and STY was calculated. In Test 1, the flow rates of the aqueous and organic phases were 6 μL/min and 2 μL/min respectively, and the aqueous-to-organic phase ratio was 3:1. In Test 2, the flow rates of the aqueous and organic phases were 18 μL/min and 6 μL/min respectively, and the aqueous-to-organic phase ratio was kept at 3:1. In Test 3, the flow rates of the aqueous and organic phases were 16 μL/min and 8 μL/min respectively, and the aqueous-to-organic phase ratio was 2:1. Finally, in Test 4, the flow rates of the aqueous and organic phases were kept at 12 μL/min and 12 μL/min respectively, and the aqueous-to-organic phase ratio was 1:1. Due to the fact that the total flow rate in Test 2, 3, and 4 was three times that of Test 1, the reactor length (the length of the outlet tube) was also increased 3-fold to achieve an equivalent reaction time of 30 s. As seen in the microscopy images embedded in Table 1, when the overall flow rate of the system was increased (from Test 1 to Test 2), the diameter of the microdroplets decreased, only one microdroplet size was generated and the number of microdroplets increased. Using the pipeline described in Section 2.5 and SI we calculated a slightly increased total interfacial area in Test 2, which also corresponds with a slight increase in STY. As the percentage of organic phase increased (Test 3 to Test 4), the average diameter of microdroplets also increased, as the number of generated droplets and the interfacial area between the two phases. For the total interfacial area shown in Table 1 and in subsequent calculations, the interfacial area calculated from Test 2, 3, and 4 is considered to be three times the interfacial area calculated from a single field of view, as the total flow rates were three times that of Test 1. Although the difference in STY between the tests is small, a reproducible trend can be identified correlating a higher total interfacial area with a higher STY.

**Table 1:**
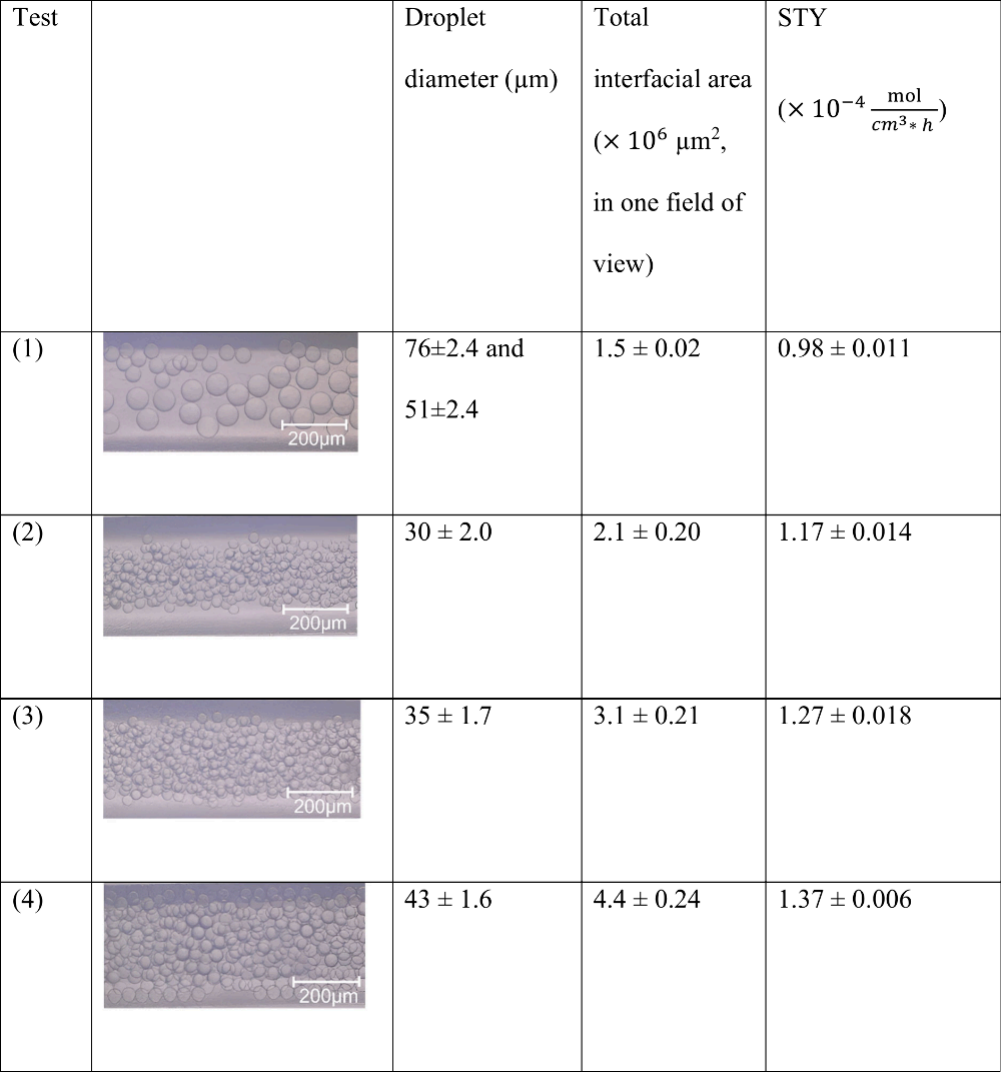
STY for Different Microdroplet Sizes, Size Distribution, and Population^a^

a In all cases, the reaction time was 30 s, accounting for time spent in the microfluidic device and tubing. In Test 1, the flow rates of the aqueous and organic phases were 6 μL/min and 2 μL/min, respectively. The aqueous-to-organic phase ratio was 3:1. In all subsequent experiments, the total flow rate was kept at 24 μL/min, but the ratio of aqueous to organic phase was changed as follows: 3:1 in Test 2, 2:1 in Test 3, and 1:1 in Test 4. Distributions derived from at least 3 experimental replicates.

**Figure 7:**
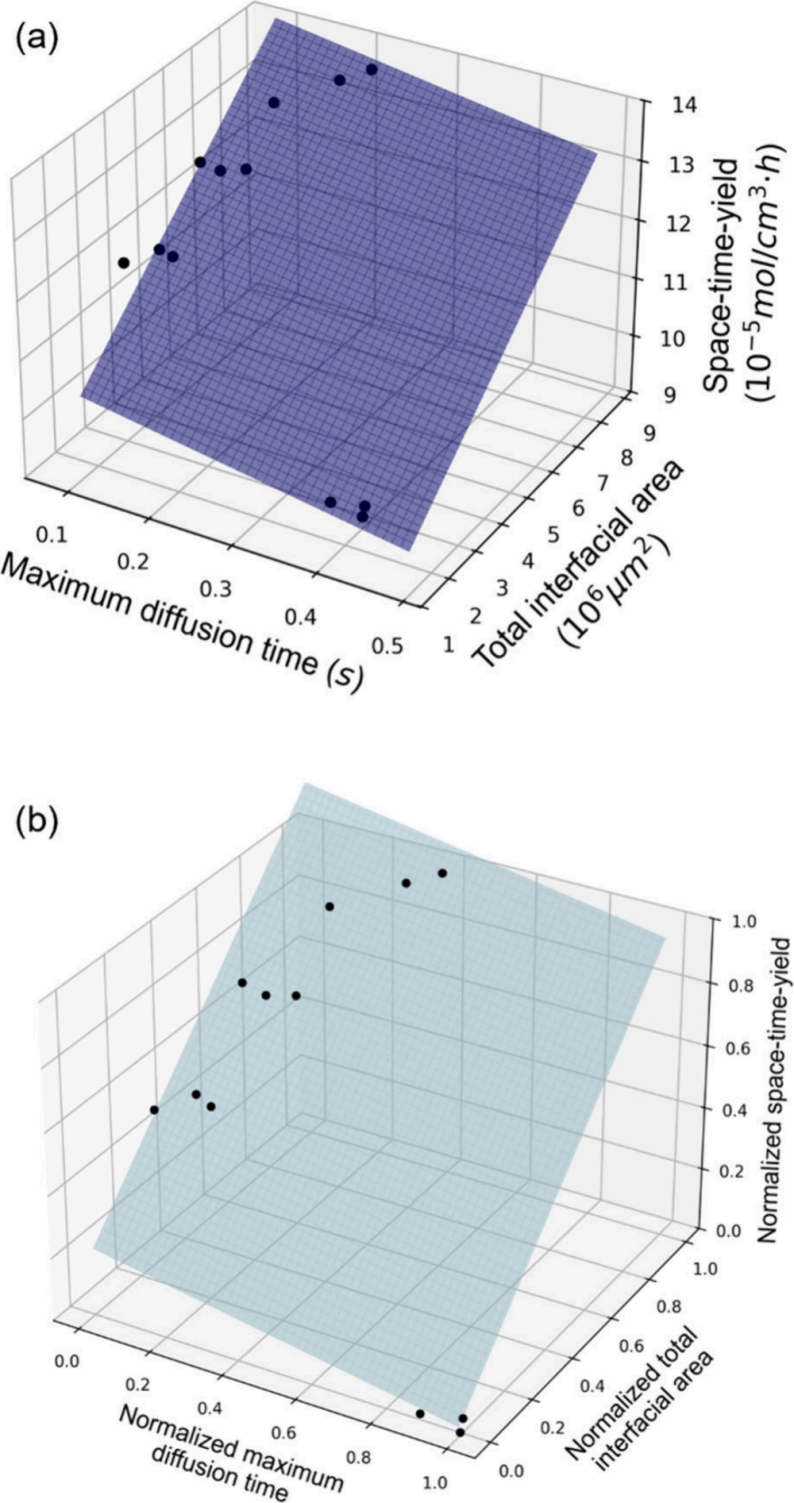
STY of microdroplets in relation to diffusion time and total interface area. (a) Multivariate linear regression derived via fitting raw experimental data (green dots). The fitted plane is described by the expression , R^2^ = 0.938. An increase in interfacial area results in a steep increase in STY, while an increase in diffusion time results in a slight decrease in STY. (b) Normalized multivariate linear regression. The fitted plane is described by the expression STY = 0.95A-0.20*t*_*max*_+0.22, R^2^=0.938. The effects of maximum diffusion time and interfacial area on STY can be compared by comparing their respective coefficients.

Throughout the discussion above, we hypothesized that larger droplet diameters would translate to longer diffusion paths to the liquid–liquid interface and lower STY. Similarly, we have seen that a larger interfacial area is correlated with higher STY. To confirm our findings and substantiate our hypotheses, we employed mathematical modeling. Starting with diffusion, maximum diffusion time is defined as the time for a substrate molecule to diffuse from the center of the microdroplet to the surface of the microdroplet, and was calculated according to the Stokes–Einstein equation and Fick’s second law as follows:^54^
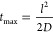
1
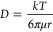
2where *t*_*max*_ is the maximum diffusion time, *l* the droplet radius and also the diffusion path, *D* the diffusion coefficient, *k* the Boltzmann constant , T the reaction temperature (here T = 25 °C = 298.15*K*), μ the solvent dynamic viscosity (for TBME μ = 0.36 *cp* (mPa*s)), and *r* is the hydrodynamic radius. For testosterone, we have approximated *r* to be equal to the molecular radius (r = 5.05 × 10^–10^) of the molecule, as shown in Figure S11. We believe that this approximation is reasonable given that testosterone is dissolved in TBME, and TBME does not form strong solvation shells around hydrophobic molecules like testosterone due to the minimal specific interactions (like hydrogen bonding or dipole–dipole interactions). Equation 1 is used for nonsteady-state diffusion, as the substrate concentration gradient in each droplet changes over time as substrate is catalyzed at the droplet boundary. Using the maximum diffusion time and total interfacial area as independent variables, a mathematical model was developed that predicts their effect on STY. A multivariate linear regression fit to the data shown in Figure 7a was performed using Scipy (leastsq() function) to obtain a three-dimensional plane, resulting in the following empirical equation:
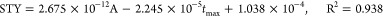
3where *A* is the total interfacial area. The generated three-dimensional plane can be used to estimate STY for a given maximum diffusion time and interfacial area. The model is based on four independent experimental sets, each performed in triplicate, ensuring the reliability of the observed trends. The high goodness of fit (R^2^ = 0.938) further supports the robustness of the model for the given experimental data sets. Looking at the coefficients from eq 3 above, one might be tempted to conclude that the effect of interfacial area on STY is much weaker than the effect of diffusion time. However, this is not accurate, as the units for these quantities are different. Looking at the graph in Figure 7a one can see that an increase in interfacial area results in a steep increase in STY, while a decrease in diffusion time results only in a slight increase in STY. Next, data normalization was performed to compare the weighted effects of maximum diffusion time and interfacial area on STY more directly and unit-independent (Figure 7b). Normalization is implemented via the MinMaxScaler in python, which processes parameters in each individual dimension into a value between [0, 1]. The normalized expression is given by
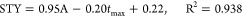
4

Looking at the normalized coefficients measuring maximum diffusion time and total interfacial area, we can see that total interfacial area has a larger and positive effect on STY, whereas diffusion time has a smaller negative effect. In practice, however, the total interfacial area and maximum diffusion time are not completely independent variables. For example, as the organic phase fraction rises, the microdroplets size increases, leading to both an extended maximum diffusion time and a larger total interfacial area. While the trend observed here is consistent across the replicates, as they were taken into account for the derivation of these equations, the equations themselves would not be transferable to other reactions, i.e., the coefficients would change based on the experimental results. However, given a maximum diffusion time and total interfacial area for other reactions, the same methods used to derive these equations could be applied to develop a mathematical model that predicts their effect on STY in a similar manner.

## 4 Conclusions and Future Perspectives

In this work, we sought to investigate biphasic biocatalysis in microfluidic droplets and systematically characterize the effect of important experimental parameters on reaction outcomes. For that, biocatalytic testosterone dehydrogenation was performed in batch, microdroplet flow, and microslug flow, and reaction outcomes were compared in terms of STY. This metric was chosen as it accounts for differences in reaction time and reaction volume between batch and microflow reactions. Overall, STY of reactions performed in microfluidics surpassed that of reactions performed in batch when using higher organic phase proportions and higher substrate concentrations. The better performance of microfluidic reactions was credited to shorter diffusion paths and larger interfacial areas available for the reaction. Although both microfluidic droplet systems performed better than batch configurations, reactions performed in microdroplet flow resulted in overall higher STY at higher organic phase ratios compared to the commonly used slug flow. Our investigations revealed a strong correlation between experimental conditions and droplet size in both microdroplet flow and slug flow. This correlation was then identified as the driving force behind differences observed in reaction performance, as droplet size dictates both the available interfacial area for the reaction and the diffusion path that the substrate (contained in the droplet) needs to travel to reach the enzyme. As the diameter of microdroplets is significantly smaller than that of slugs, the substrate diffusion path is smaller, and therefore, the reaction rate is higher. Similarly, as microdroplets do not generally touch the channel walls and are only in point contact with each other, a larger interfacial area was found to be available for the reaction compared to slugs.

Although it might seem intuitive that a large interfacial area between the aqueous and organic phases could increase the productivity of the reaction, a higher contact area between the two phases has also been reported to lead to increased enzyme inactivation, and therefore can negatively affect the outcome of the reaction.^55^ In this work, we show that in microdroplet flow the potentially negative effect of enzyme inactivation upon increased interfacial area was negligible compared to the positive effects arising from shorter diffusion paths and increased reaction surface. This certainly depends on the solvent system used and the enzyme employed. Although only testing our microdroplet platform for reactions using CtHSD in this work, we can foresee similar outcomes for reactions utilizing other enzymes such as lipases, glucose oxidases, and catalases, which have also demonstrated higher reaction rates and stability in microfluidic biphasic systems.^36,56,57^

The negligible enzyme inactivation upon increased interfacial area could also be related to the use of surfactants. As surfactants adsorb at the interface between the aqueous and organic phases, they could sterically reduce the contact between the enzyme molecules and the organic phase, thereby decreasing enzyme inactivation. In general, we observed that adding a small amount of surfactant to all reaction systems, including batch reactions, boosted STY. The exact mechanism behind this boost cannot be easily deconvoluted as the addition of surfactants triggers a cascade of physical changes in the system, including changes in slug lengths and stabilization of bimodal microdroplet sizes at low flow rates, which in turn change the total available reaction interface and overall substrate diffusion paths.

In conclusion, our work offers evidence of the potential of microdroplet flow as a reaction system for biphasic biocatalysis and provides first insights into the mechanisms that drive an increase in STY compared to the more commonly used batch and slug flow configurations. The potential trade-off between maximized mass transfer and possibly higher enzyme inactivation rates in small droplets with large surface-to-volume ratios were also investigated for the first time and a pipeline was established for the calculation of interfacial areas that can be used for similar evaluations in other reactions. Finally, the effect of the use of surfactant on STY was investigated for the first time, which could be used as expectation guidance when surfactants are added to a two-phase biocatalytic reaction.

As we delve into the mechanisms behind STY, we provide researchers with the tool kit necessary to make informed decisions about the most suitable technology to use for their reactions and we demonstrate the advantages of using droplet microfluidics compared to batch reactions in two-phase biocatalysis. Further research could focus on optimizing microdroplet systems for industrial scalability, examining the long-term stability of enzymes in these systems, and exploring a wider range of biocatalytic reactions. However, even in the absence of scalable microfluidic technologies for industrial-level production, we have demonstrated that microfluidic experiments can offer valuable insights owing to precise experimental control, which can be used for process understanding and reaction optimization even at the larger scales. When straightforward scaling up is the primary objective, other methods of generating monodisperse droplets in continuous flow systems could be more relevant, primarily membrane emulsification.^58,59^ However, microfluidics generally offers better control over droplet size, size distribution, and stability, which can enhance reaction kinetics and mass transfer, especially in biocatalytic processes. Ultimately, a decision related to the most suitable droplet forming technology should depend on the desired droplet characteristics, reaction conditions, and scalability requirements. Finally, future studies could investigate the effects of different surfactants and solvent systems in greater detail, providing deeper understanding into their roles in enhancing reaction outcomes.

## Supporting Information Available

Experimental details related to gene cloning, enzyme production and purification, determination of reaction equilibrium, optimal enzyme concentration, enzymatic half-lives, microfluidic droplet formation, experimental setup for two-phase biocatalysis in batch and in microfluidics, microfluidic device fabrication, microfluidic droplet detection for interfacial area calculations, kinematic viscosities test, drop formation junction, impact of Tween 20 on CtHSD activity, testosterone partition coefficient, residual enzyme activity determination after the two-phase reactions, and molecular model of testosterone (PDF)
