# Supplementary material for: Evaluating the Potential of Microdroplet Flow in Two-Phase Biocatalysis: A Systematic Study
Source: ACS Appl Mater Interfaces. 2025 Jan 10;17(3):4776–87. doi: 10.1021/acsami.4c15647 (PMC11758778; doi:10.1021/acsami.4c15647)
Supplement: Supplementary file 1 — am4c15647_si_001.pdf [file am4c15647_si_001.pdf]

Supporting Information

Evaluating the Potential of Microdroplet Flow in Two-Phase Biocatalysis: A Systematic Study

Lanting Xiang[a,b], *Jennifer Solarczek*[c], *Victor Krajka*[a,b], *Hui Liu*[d], *Lina Ahlborn*[c], *Anett Schallmey*[b,c], *Iordania Constantinou*[a,b,*]

[a] Institute of Microtechnology (IMT), Technische Universität Braunschweig, Alte Salzdahlumer Straße 203, DE-38124, Braunschweig, Germany

[b] Center of Pharmaceutical Engineering (PVZ), Technische Universität Braunschweig, Franz-Liszt-Str. 35a, DE-38106, Braunschweig, Germany

[c] Institute for Biochemistry, Biotechnology and Bioinformatics, Technische Universität Braunschweig, Spielmannstr. 7, DE-38106, Braunschweig, Germany

[d] Cognitive Systems Lab (CSL), University of Bremen, Enrique-Schmidt-Str. 5, DE-28359, Bremen, Germany

*Email: i.constantinou@tu-braunschweig.de

Experimental details

Gene cloning

The synthetic gene of the 3β/17β-hydroxysteroid dehydrogenase from Comamonas testosteroni (CtHSD; GenBank accession number: WP_003080542.1) was ordered as gene fragment with codon optimization for Escherichia coli. Using the Golden Gate Assembly method and BsaI-HF®v2 as endonuclease, the gene was cloned into the pET28a(+) expression vector, which is providing an N-terminal His-tag to the enzyme.

Enzyme production and purification

CtHSD was produced in E. coli BL21 (DE3) Gold cells from vector pET28a(+), which allows for recombinant expression of the gene via the T7 expression system. For this, 500 mL TB medium containing 50 µg/mL kanamycin was inoculated with 5 mL of the respective pre-culture and incubated at 37 ˚C until an OD600 between 0.5 and 0.6 was reached. Then, expression was induced using 0.5 mM isopropyl β-D-1-thiogalactopyranoside (IPTG), and the culture was further incubated at 22 ˚C for 22 h. Afterwards, the cells were harvested by centrifugation (3880 g, 20 min, 4 ˚C) and stored at -20 ˚C until further use. Purification of the His-tagged CtHSD was performed using immobilized metal ion affinity chromatography (IMAC) and a 5 mL HisTrapTM FF column connected to an Äkta pure FPLC system. The respective frozen cell pellet was re-suspended in buffer A (20 mM Tris, 500 mM NaCl, 20 mM imidazole, 5 mM ß-mercaptoethanol, pH 7.9) containing 1 mg/mL lysozyme and one protease inhibitor cocktail tablet (Thermo Fisher

Scientific, Waltham, USA) per 10 mL buffer A. The cells were disrupted by sonication (5 min of 10 s pulses at 65% amplitude and 20 s pause, on ice). After centrifugation (16600 g, 30 min, 4 ˚C), the obtained cell-free extract was filtered (0.45 μm cellulose acetate membrane filter) and loaded on the HisTrapTM FF column equilibrated with buffer A. The bound proteins were eluted using a 80 mL linear gradient from 20 mM to 500 mM imidazole and the elution was monitored at 280 nm. CtHSD-containing fractions of the elution were pooled, concentrated to a volume of 2.5 mL, and desalted using a PD-10 column and storage buffer (20 mM Tris, 50 mM NaCl, 1 mM EDTA, 1 mM DTT, 20% glycerol, pH 8). The protein solution was aliquoted à 50 µL and stored at -20 ˚C until further use. Yields of purified CtHSD typically exceeded 400 mg/L expression culture.

Determination of the reaction equilibrium

To determine the equilibrium constant of the CtHSD-catalyzed testosterone dehydrogenation, 200 µL reactions containing 20 mM Tris-HCl buffer, 25 mM NaCl, pH 9, 50 µg/mL CtHSD, 200 µM NAD+ and 0-4 eq testosterone were performed in a microtiter plate at room temperature. Reactions were followed spectrophotometrically in a microplate reader (Clariostar, BMG Labtech) by measuring the absorbance of formed NADH at 340 nm over 11 min (to reach the reaction equilibrium in each case). The resulting A_{340} absorbance values in the equilibrium states were translated into conversion values for the conversion of NAD+ to NADH via the extinction coefficient of NADH at 340 nm under the applied reaction conditions (5.7 mM-1 cm-1, measured using a 200 µM solution of commercial NAD+ under the reaction conditions described above) using equation (**Equation S1**).

$$c_{eq} = \frac{A_{340,eq} - b}{\varepsilon\gamma}$$ (**Equation S1**)

where c_{eq} is the extent of conversion in the equilibrium (in fractions, not %), $A_{340,eq}$ is the absorbance value at 340 nm in the equilibrium, b is the background absorbance (0.087 in our setup), ε is the extinction coefficient of NADH at 340 nm (5.7 mM-1 cm-1) and γ is the initial concentration of NAD+ (200 µM in our case). The resulting equilibrium conversions were plotted against the applied testosterone equivalents (Figure S1) and fitted according to equation (**Equation S2**), which is derived from the law of mass action, as described recently [1,2].

$$c_{eq} = \frac{-K - Kx + \sqrt{(K+Kx)^2 + 4Kx(1-K)}}{2-2K}$$ (**Equation S2**)

where K is the equilibrium constant of the transformation and x is the excess of substrate (testosterone in this case; i.e. equivalents of testosterone over NAD+). All these variables are dimensionless. This fit yielded K = 2.18 ± 0.46 (R2 = 0.981) as an estimate of the equilibrium constant.

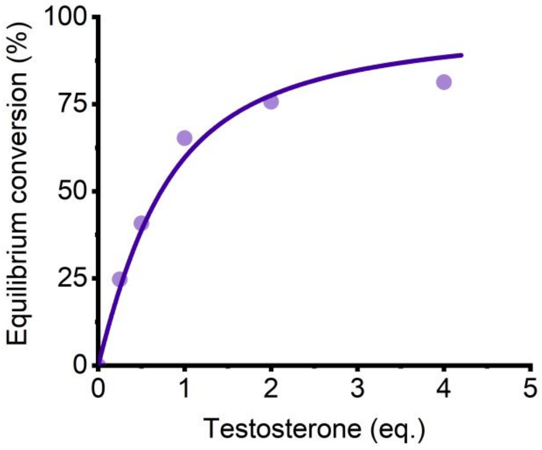

Figure S1. Oxidation of testosterone using 50 µg/mL CtHSD using a fixed NAD^+ concentration (200 µM) and different equivalents of testosterone. Conversion is defined as the percentage of substrate (here NAD^+) that has been converted to product.

Determination of optimal enzyme concentration

To evaluate the optimal enzyme concentration to be used in two-phase biocatalytic reactions, batch reactions were performed in glass vials in a total reaction volume of 500 µL using three different CtHSD concentrations (10, 20 and 50 µg/mL) at 10% (v/v) organic phase proportion (**Figure S2**). This revealed reactions containing 50 µg/mL CtHSD to yield significant conversion already after 2 min reaction time while still being far away from the reaction equilibrium.

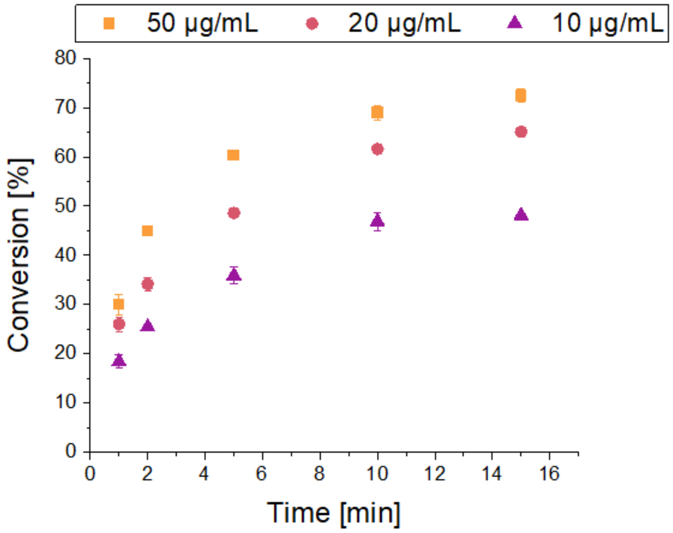

Figure S2. Conversion of testosterone in two-phase biocatalysis in batch at varying enzyme (CtHSD) concentration. Reactions were performed in 20 mM Tris-HCl, 25 mM NaCl, pH 9 containing 2.5 mM testosterone, 5 mM NAD^+ and 10% TBME at 25˚C and 900 rpm. Reactions were performed in triplicate and analyzed by HPLC. Error bars show the standard deviation.

Determination of enzymatic half-lives

For the determination of enzymatic half-lives in the presence of *tert*-butylmethylether (TBME) or diisopropylether (DiPE), the CtHSD (20 µg/mL) was incubated in 20 mM Tris-HCl buffer, 25 mM NaCl, pH 9, containing 10% of the respective solvent at 25°C and 900 rpm shaking. After 0, 1, 2, 3, 4, 5, and 6 min (TBME) or 0, 1, 2, 3, 4, 5, 6, 9, and 16 min (DiPE), each 50 µL samples were taken and mixed with 50 µL reaction mixture containing 20 mM Tris-HCl buffer, 25 mM NaCl, pH 9, 2 mM NAD^+, and 1 mM testosterone (from a stock dissolved in 36% (w/v) 2-hydroxypropyl-β-cyclodextrin in H_2O). Each reaction was followed spectrophotometrically by measuring the absorbance of formed NADH at 340 nm and room temperature. Resulting enzyme activities were calculated from the respective absorbance increase according to the law of Lambert-Beer using a molar extinction coefficient ε for NADH of 6300 M^{-1} cm^{-1}.

Activities were plotted against incubation time (**Figure S3**) and fitted as a first-order exponential decay according to equation (**Equation S3**), yielding estimated half-lives via equation (**Equation S4**).

$$a(t_{inc}) = a_0 \exp\left(-\frac{t_{inc}}{\tau}\right)$$ (**Equation S3**)

$$t_{1/2} = \tau\, ln(2)$$ (**Equation S4**)

where $a(t_{inc})$ is the observed activity of the CtHSD (in mU) as a function of incubation time t_{inc} (in min), a_0 is the observed activity at the start of the incubation (in mU, where $t_{inc} = 0$ min), τ is the mean lifetime of the enzyme (in min) and $t_{1/2}$ is the half-life (in min).

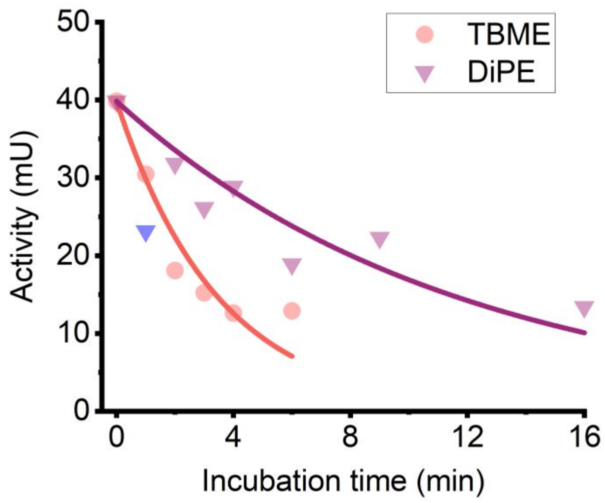

Figure S3. Activity of CtHSD after incubation in 10% TBME or DiPE for different time points. The same amount of enzyme was used in both solvents. The blue triangle belongs to the measurement with DiPE as solvent but was not included in the corresponding fit ("outlier").

Microfluidic droplet formation

Microfluidic droplets can be produced in a variety of shapes and sizes based on the formation method used and the geometry of the microfluidic device. When microfluidic droplets are used as microreactors, their geometry can affect the productivity of the reactions performed. In literature, microfluidic droplets formed using T-, Y- and Ψ-shaped microfluidic junctions are most commonly used for two-phase biocatalysis [3]. In these device architectures, droplets are formed when two immiscible liquids meet at an intersection. **Figure S4a** shows the design of T-junction

microfluidics used in this work and an illustration of microfluidic droplet formation. Under the action of pressure and shear, the continuous phase (here the aqueous phase in blue) cuts off the dispersed phase (here the organic phase in yellow) and droplets are formed. Depending on the dimensions of the microchannel and the flow parameters, the T-junction design can produce either large, ellipsoidal droplets (often referred to as "slugs") or smaller, spherical microdroplets. In our device, the channel dimensions favor the formation of relatively large slugs with widths approximately equal to the channel width (as shown in **Figure S4b**). The length of the droplets is determined by a combination of parameters including the total flow rate of the system and the phase ratio, which in microfluidic systems is determined by the flow rate ratio of the two phases. In this configuration, although the slugs are separated from the microchannel walls by a thin film of the continuous phase, the film's volume is negligible [4] and thus, the only practically available interfaces between the aqueous and organic phases that can host the enzyme-catalyzed reaction are the two ends of the slugs.

Although not commonly used in two-phase biocatalysis, flow-focusing microfluidic architectures are also used to generate microfluidic droplets (**Figure S4c**). In this architecture, the continuous phase is introduced into the microfluidic device through two side channels, which are used to squeeze the dispersed phase flowing through the central channel [3]. Spherical microdroplets are generated when the dispersed phase flows through a narrow neck at the entrance of the central channel. These microdroplets are generally much smaller than the width of the central channel (**Figure S4d**), with typical diameters on the order of tens of micrometers, and they tend to flow in the middle of the channel. As microdroplets are not in contact with the channel walls and are

separated from each due to steric hinderance resulting from the use of surfactant (see **Section 3.1**), a larger overall interfacial area is available for the biocatalytic reaction. A more detailed discussion about interfacial area is provided in the discussion section below along with relevant experimental results.

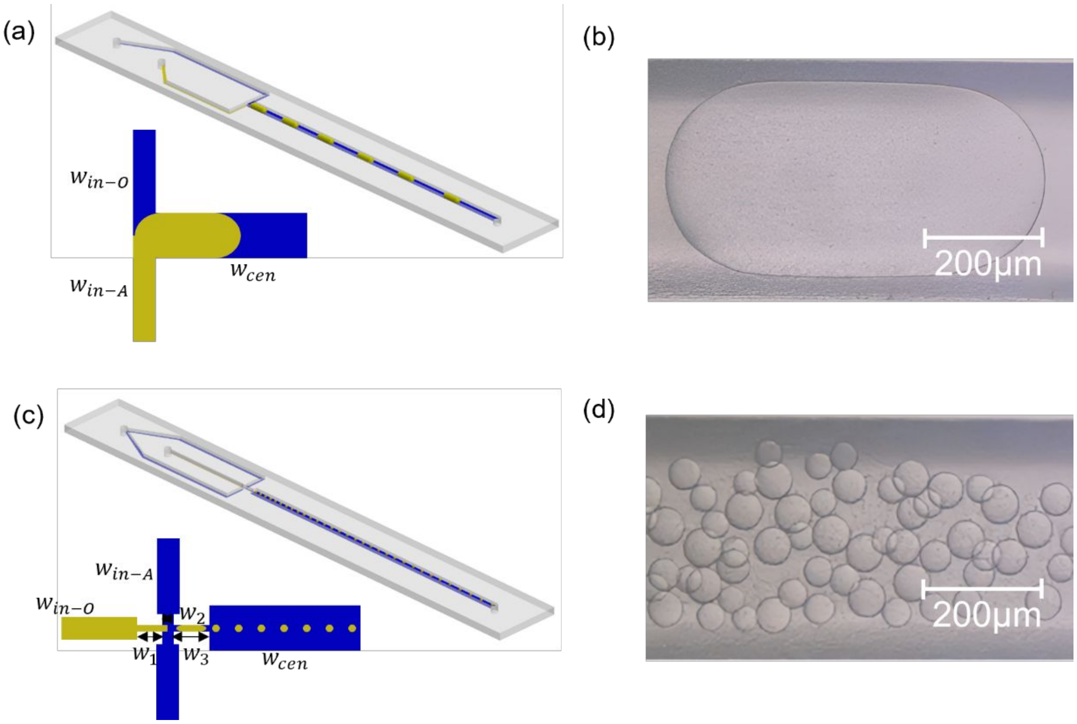

Figure S4. (a) T-junction device architecture and depiction of microdroplet generation and flow; (b) Digital microscope image of microfluidic slugs generated in a flow-focusing system; (c) Flow-focusing device architecture and depiction of slug generation and flow; (d) Digital microscope image of microfluidic droplets generated in a T-junction. The central channel of both microfluidic devices used herein has a length of 21770 μm, a width of 400 μm (w_{cen}), and a height of 100 μm. The inlet channel width for both the aqueous and organic phases (w_{in-A}, w_{in-O}) in both

microfluidic devices is 200 μm. The cross-sectional widths of the flow-focusing device are as follows: w_1 = 50 μm, w_2 = 110 μm and w_3 = 60 μm.

Experimental setup for two-phase biocatalysis in batch and in microfluidics

Batch reactions were generally performed in 2.0 mL glass vials in a total reaction volume of 500 μL containing 20 mM Tris-HCl buffer, 25 mM NaCl, pH 9 and 50 μg/mL CtHSD at 25°C. Reactions were shaken at 900 rpm in a ThermoMixer C (Eppendorf) to encourage mixing and increase the contact area between the two phases. In reactions with varying Tween 20 concentration (0-5%), 2.5 mM testosterone, 10% (v/v) TBME and 5 mM NAD^+ were used. Reactions were performed in triplicate and stopped after 2 min to ensure no negative impact of enzyme stability on the conversion. Reactions with varying organic phase ratio (10, 20 and 25% v/v) contained 2.5 mM testosterone and 5 mM NAD^+.

For reactions with increasing substrate concentration (1, 2, 3, 4, 5, 7.5 and 10 mM testosterone), the NAD^+ concentration and organic phase (TBME) proportion had to be adjusted relative to the testosterone concentration. For 2, 4, 6, 8 and 10 mM NAD^+, 10% TBME were applied in reactions with 1, 2, 3, 4 and 5 mM testosterone, respectively, while 10 mM NAD^+ and 15 % TMBE were used in reactions with 7.5 mM testosterone, and 10 mM NAD^+ and 20% TBME were used in reactions with 10 mM testosterone. Reactions with increasing substrate concentrations were performed in duplicate and stopped after 10 min to allow enough time for conversion.

In general, reactions were stopped by addition of 100 µL 10% *sulfuric acid* (H_2SO_4) and product was extracted with 600 µL ethyl acetate. H_2SO_4 was used to promptly deactivate the enzyme and stop the reaction [5,6]. After vigorous vortexing and short centrifugation (1 min, max. speed; MICRO STAR 17, VWR) for quick phase separation, the upper organic phase containing the product and any unconverted substrate was transferred to a new vial, and the organic solvent was evaporated by heating at 50˚C in a ThermoMixer C (Eppendorf). The residue was dissolved in 100 µL acetonitrile (ACN) and was used for subsequent high-performance liquid chromatography (HPLC) to measure the amount of residual testosterone and formed androstenedione in order to calculate conversions and STY. HPLC analysis was performed on a Prominence HPLC (Shimadzu) equipped with a Nucleoshell RP18 column (Macherey-Nagel) at 40˚C. The presence of androstenedione was confirmed by comparing the retention time of the product formed in our reactions on HPLC to an authentic commercial standard. A mixture of ACN and H_2O was used as a mobile phase with a flow rate of 0.7 mL/min starting with a 0.2 min pre-rinsing step at 20% (v/v) ACN, followed by a linear gradient from 20 to 50% (v/v) ACN over 5 min and another 0.3 min at 50% (v/v) ACN. Afterwards, the ACN concentration was reduced again to 20% (v/v) within 0.5 min with a final hold at 20% (v/v) ACN for 2 min. Detection of substrate and product was performed by UV absorbance at 254 nm. The substrate testosterone and the corresponding product androstendione were eluted at retention times of 6.2 min and 6.8 min, respectively.

Figure S5 shows the complete experimental process for two-phase biocatalysis in microfluidic droplets, including sample analysis off chip. First, the organic and aqueous phases were injected into their respective device inlets using syringe pumps (NEMESYS Base 120 + low-pressure

module). Microfluidic devices were placed on a 3D microscope (Keyence VHX-5000) in order to observe microdroplet/slug generation, monitor the microfluidic droplet characteristics (e.g. size, shape), and ensure stable flow through the device. The reaction started when the two phases met at the microchannel intersection and ended when the two phases exited the device through a shared outlet and were collected in a microcentrifuge tube, which contained the 10% H_2SO_4 solution. Thus, the total reaction time was defined as the time the two phases spent traveling through the microchannel and the outlet teflon tube (Techlab, inner diameter 0.25 mm). The reaction was performed at room temperature. Once a predefined volume of reaction solution was collected (250 µL), a base (141 µL, 2.55 M NaOH and 100 mM Na_2HPO_4 mixing solution) was added to neutralize the solution and ethyl acetate (EtOAc, 500 µL) was used to extract the complete mixture for further analysis. After vigorous vortexing, centrifugation (1 min, max. speed; MICRO STAR 17, VWR) of the total reaction solution enhanced the phase separation. The upper phase (EtOAc + TBME containing the product and any unconverted substrate) was transferred to a fresh glass vial and the solvent was evaporated as described above. The dry residue was dissolved in 100 µL ACN and analyzed via HPLC as described above.

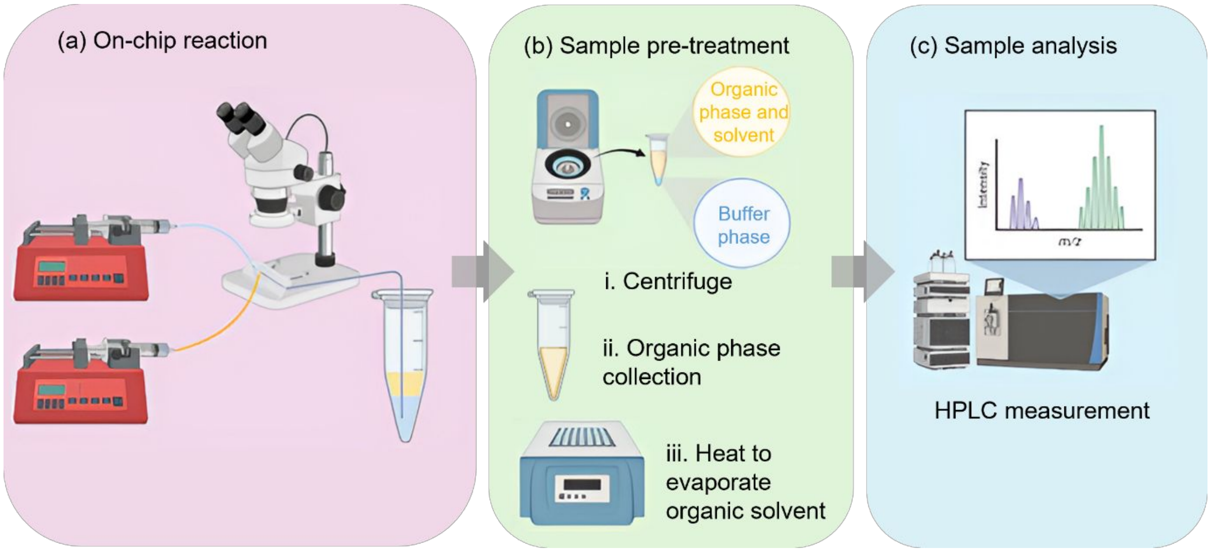

Figure S5. Experimental setup for microfluidic system. a) Microfluidic reaction and sample collection. The organic (yellow) and aqueous (blue) phases containing the substrate and enzyme respectively are introduced into the device using syringe pumps. Reaction occurs in microfluidic devices placed on a microscope for optical process monitoring. All device contents are collected in a microcentrifuge tube. b) Sample pre-treatment process. i. Centrifugation for phase separation. ii. Organic phase collection for further analysis. iii. Heating to evaporate organic solvent and obtain crude reaction product. c) Sample analysis using HPLC. Using HPLC data and known experimental conditions system, STY can be calculated.

Microfluidic device fabrication

Two microfluidic devices were designed to generate slug flow and microdroplet flow for two-phase biocatalysis on chip. Borosilicate glass wafer (G-materials e. K.) with a thickness of 700 μm was chosen as the microfluidic device material because of its high transparency and strong biochemical inertness [7,8]. The microfluidic devices were fabricated based on methods developed

and reported by Erfle et al. [9]. A *ytterbium-doped potassium tungstate* (YB:KGW) femtosecond laser with a primary wavelength of 1030 nm was used to ablate the microchannel structures on glass. The laser produced an average power of 15 W, a pulse frequency of 600 kHz, and a pulse duration of 212 fs. The laser was focused onto a spot with a diameter of 18 μm through a lens with a focal length of 100 mm. The pulse energy of the laser spot was approximately 16 μJ. The ablation process was achieved by moving the laser spot with a speed of 2000 mm/s for better surface quality. A six-layer excision pattern consisting of parallel scanning lines was set in order to achieve complete elimination. The distance between the parallel lines and the offset from the channel edges was set to 4 μm and each scan layer was rotated by 30° thus achieving a full 360° scene scan. Femtosecond laser ablation is followed by the pre-bonding process. The pre-bonding process (**Figure S6b**) typically involves several key stages that ensure the surfaces to be bonded (in this case a structured glass wafer that contains the microfluidic channels and a sealing glass wafer) are conditioned to improve surface characteristics, optimize adhesion, and eliminate any contaminants that could interfere with the final bonding process. Firstly, glass wafers were immersed in a hydrofluoric acid solution (de-ionized water, phosphoric acid (85%), hydrofluoric acid (40%)) to etch (clean) glass particles remaining on the structured surfaces and in microchannels. Borosilicate glass wafers were then cleaned in an ultrasonic water bath for 20 minutes and were placed in piranha solution (*sulfuric acid* (H_2SO_4)), concentrated, *hydrogen peroxide* (H_2O_2, 30%); 2:1) for deep cleaning and to increase the hydrophilicity of the surface. A clean blank glass wafer was used as the sealing layer. The pre-bonded glass wafers were then placed in a muffle furnace (Laborofen

VMK - 135 S Sonder, Linn High Therm GmbH) at 655˚C for 6 hours to thermally bond. The final step of the fabrication process is heating to 750˚C for 1 hour to achieve minimum surface roughness and the best surface quality. **Figure S6** shows the complete microfluidic device fabrication process.

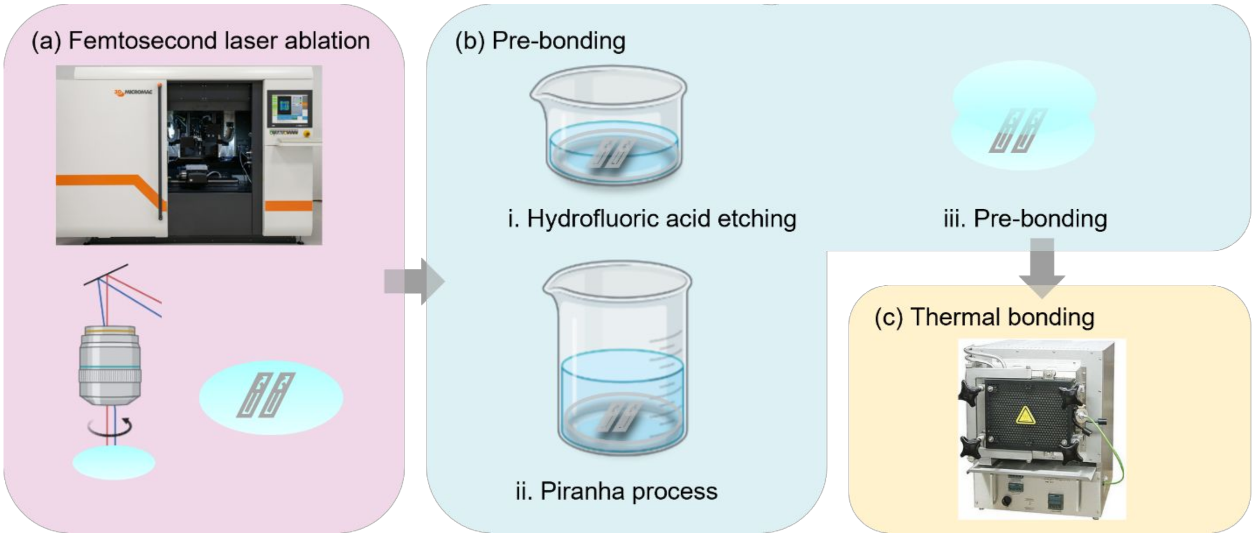

Figure S6. Microfluidic device fabrication process. a) Femtosecond laser ablation, channel formation on glass wafer. The schematic depicts a femtosecond laser instrument used for microstructuring through ablation. Laser light focused through an objective lens ablates structures on a glass wafer. b) Pre-bonding process. i. Hydrofluoric acid etching of ablated glass wafer, ii. piranha process, iii. pre-bonding / sealing with a blank glass wafer. c) Thermal bonding in muffle furnace to create leak-proof channels.

Microfluidic droplet detection for interfacial area calculations

As a first step to microfluidic droplet detection, microscopy images of microdroplets had to be segmented. Since the illumination in the microfluidic channel was not uniform, standard thresholding with ImageJ did not produce acceptable results. Therefore, a neural network was trained to denoise and segment the original images according to the user manual [10,11]. Briefly, training and validation images (inverted 16-bit grayscale, ~180 x 1200 pixels each) were manually segmented by drawing circles around microfluidic droplets formed at each aqueous/organic phase ratio using ImageJ (**Figure S7a**). Next, the deep learning software was trained until a validation stop-loss minimum was reached (**Figure S7b**). One epoch consisted of the following parameters: 200 steps, 64 stack sizes, stack shape 64 and neighbourhood radius 5. The trained model was employed to predict droplet segmentation of inverted 16-bit grayscale images, resulting in a denoised image and outlines of the segmented microfluidic droplets (**Figure S7c**). For the subsequent Hough Circle Transform (HCT), the search parameters were set as follows: Radius search increment 1, maximum circle score 1200, transform resolution 1000, clear neighbour radius ratio 1. The minimum/maximum search radius and the Hough score threshold depended on the droplet size and the image resolution. Finally, circular masks were superimposed on the denoised input images to verify the droplet detection efficiency (**Figure S7d**). Even in the most challenging reaction conditions to track, that produced the smallest microfluidic droplets, the tracking result was very robust (**Figure S7e**).

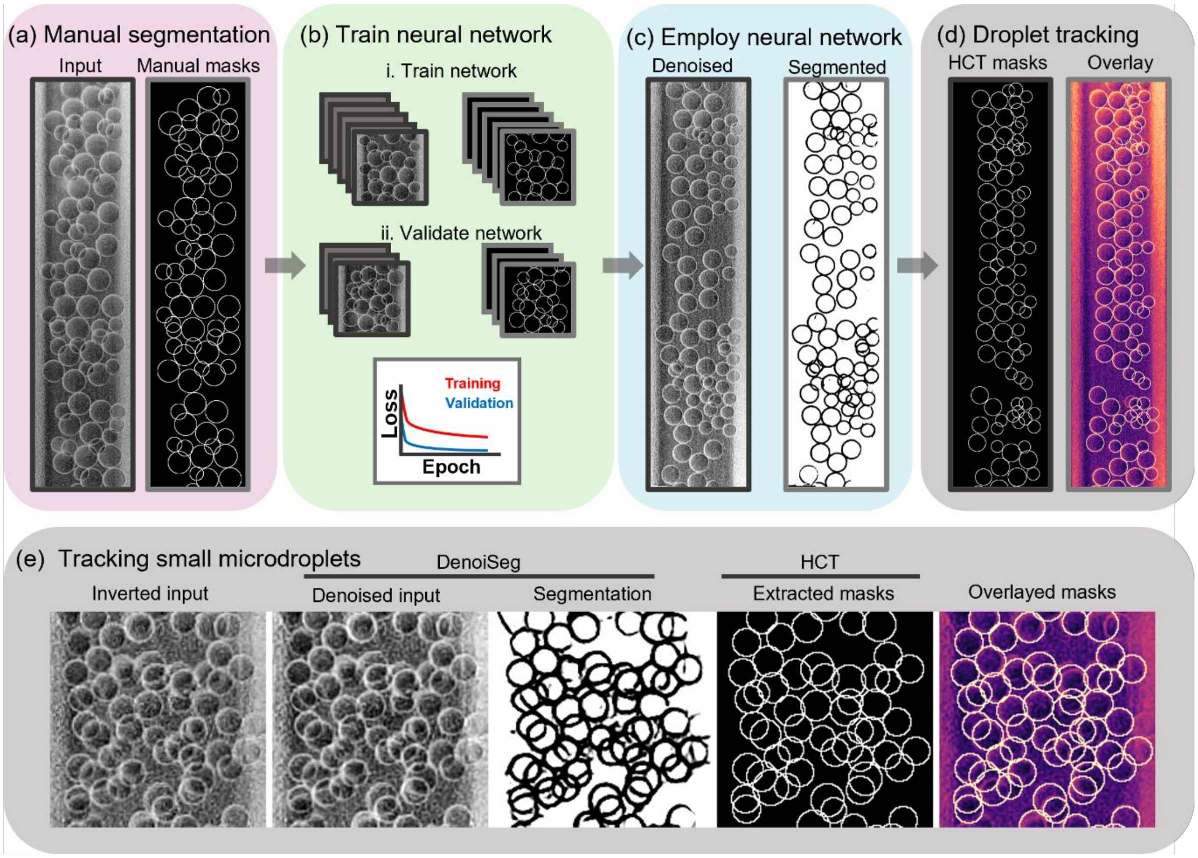

Figure S7. Droplet analysis pipeline. a) First, training and validation data sets were generated by manually assigning microfluidic droplet masks for all aqueous/organic phase conditions. b) Neural network (DenoiSeg) was trained until validation stop-loss was reached. c) Trained network was employed for image denoising and droplet segmentation. d) Microfluidic droplets were tracked by conducting *Hough Circle Transform* (HCT). The goodness of fit was verified by superimposing the generated masks on the input images (pseudo-colour magma scale). e) The detection of microfluidic droplets was reproducible under all reaction conditions. For this example, a representative image of the reaction giving the smallest microfluidic droplet sizes was chosen (aqueous phase 18 µl/min and organic phase 6 µl/min).

Based on the size of the detected microfluidic droplets, the measured circle radii were used to calculate the interfacial area (spherical area; $A = 4 \times \pi \times r^2$). The mean value of all images was calculated and normalized to the field of view to calculate the total interfacial area. The robustness of our microfluidic droplet detection pipeline we assessed by comparing with already published TrackMate detectors (**Figure S8**) [12]. Since some detectors have various sensitivities to inverted and non-inverted images, both conditions were tested. The results could be categorized into three groups. The first group of detectors could detect overlapping microfluidic droplets with uniform-size tracking masks. These included *Differences of Gaussian* (DoG), Hessian and Laplacian of Gaussian (LoG) detectors. In this group, the sensitivity was higher for non-inverted images. The MorphoLibJ and Cellpose detectors were able to detect microfluidic droplets of different sizes, but overlapping masks were not allowed [13,14]. This is because the detectors are primarily designed to detect cells that do not overlap in one plane. MorphoLibJ showed a higher sensitivity for the inverted input, while Cellpose displayed improved matching masks for the non-inverted image. Of all the Trackmate detectors, Cellpose demonstrated the most accurate detection results. The detection pipeline developed in this work and described above could consistently label overlapping and different-sized microfluidic droplets in inverted images. The false positive rate for the non-inverted image was relatively high because the neural network was trained exclusively using inverted images. A comparison table of the different detectors can be found in the supplement (**Table S1**).

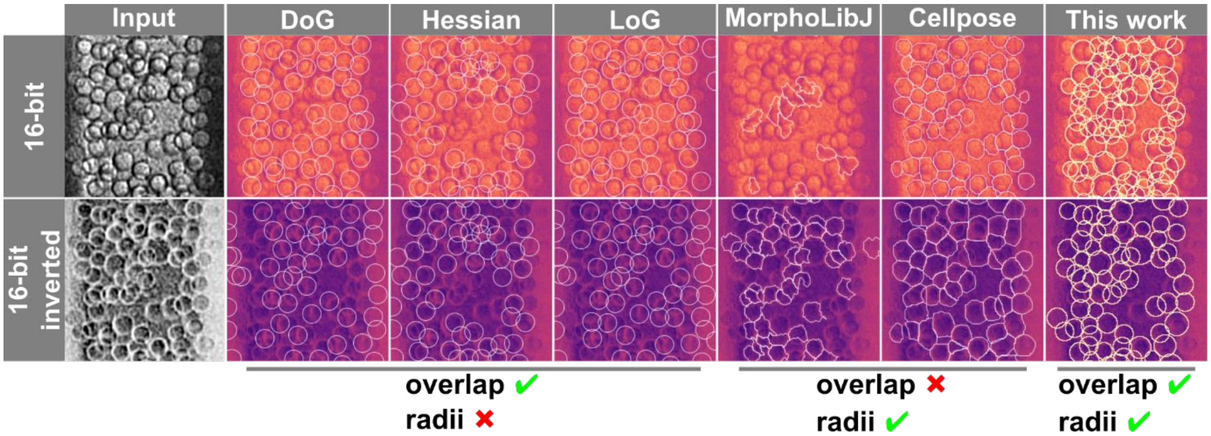

Figure S8. Comparison of advanced blob and tracking plugins for ImageJ (TrackMate) for microfluidic droplet detection. The blob detectors DoG, Hessian, and LoG could detect overlapping microdroplets but with masks of equal radius. The cell tracking detectors MorphoLibJ and Cellpose detected droplets of different radii but could not label overlapping events. Our neural network-based approach could detect microfluidic droplets of different sizes and overlapping events. The masks were overlaid on pseudo-coloured input images (magma scale) for clearer presentation. DoG: Differences of Gaussian, LoG: Laplacian of Gaussian.

Table S1. Parameter setup for representative droplet analysis

Detector	Image type	Counted droplets	Radius distribution	Overlap	False-positive	False-negative	Parameters
	16-bit grayscale						

DoG*	normal	286	uniform	yes	medium	medium	Estimated diameter: 22 pixels, quality threshold: 200, pre-process median filter: yes, sub-pixel localization: yes
	inverted	277	uniform	yes	high	high	
Hessian*	normal	359	uniform	yes	medium	high	Estimated diameter: 22 pixels, quality threshold: 150, normalize quality values: no, sub-pixel localization: yes
	inverted	359	uniform	yes	medium	high	

LoG*	normal	328	uniform	yes	mediu m	high	Estimated diameter: 22 pixels, quality threshold: 30, pre-process median filter: yes, sub-pixel localization: yes
	inverted	324	uniform	yes	high	high	
MorphoLibJ * (Legland et al. 2016)	normal	100	diverse	no	high	very high	Tolerance: 5000, connectivity: diagonal, simplify contours: yes, circularity: >0. 4, area: <430, perimeter: > 54
	inverted	299	diverse	no	low	high	

Cellpose* (Stringer et al. 2021)	normal	337	diverse	no	very low	very low	Model: Cytoplasm, Channel to segment: grayscale, optional second channel: none, cell diameter: 22 pixel
	inverted	333	diverse	no	very low	very low	
DenoiSeg (Buchholz et al. 2020) + HCT (this work)	normal	394	diverse	yes	very low	very high	Min radius: 9, Max radius: 18, Radius increment 1, Max number of circles: 1200, Hough score threshold: 0.85, Transform resolution:
	inverted	399	diverse	yes	very low	very low	

							1000, clear neighbors radius ratio: 1

*tested using Trackmate plugin (Ershov et al. 2022) for ImageJ

Kinematic viscosities test

At room temperature, the kinematic viscosities of two liquids were measured using a capillary viscometer (Model: 501 01/0a, SI Analytics GmbH, Mainz). The liquids tested were a buffer solution (20 mM Tris-HCl, 25 mM NaCl) containing no Tween 20 and the same buffer solution containing 8% Tween 20. The kinematic viscosity was calculated according to equation (**Equation S5**):

$$\nu = K \cdot t \qquad \text{(Equation S5)}$$

where ν represents the kinematic viscosity, K= 0.005016 mm^2/s is the viscometer constant, and t is the flow time. Each liquid was measured three times independently, and the average viscosity and standard deviation were calculated. The flow times for the buffer solution were 3 min 19.77 s, 3 min 21.63 s and 3 min 21.33 s, resulting in an average kinematic viscosity of 1.0078 ± 0.0041 mm^2/s. For the buffer solution containing 8% Tween 20, the flow times were 5 min 29.03 s, 5 min 31.32 s and 5 min 31.61 s, yielding an average kinematic viscosity of 1.6586 ± 0.0058 mm^2/s. These results indicate that at room temperature, the addition of 8% Tween 20 significantly

increased the kinematic viscosity of the buffer solution, reflecting the enhanced internal friction and altered flow properties caused by the presence of Tween 20.

Drop formation junction

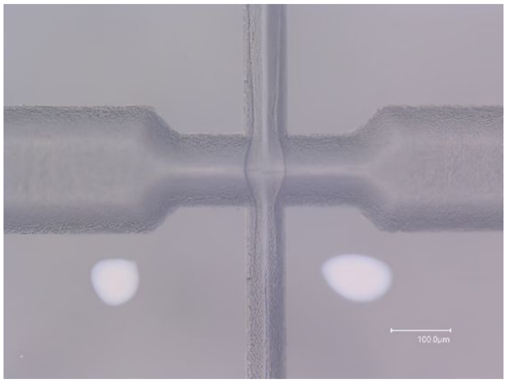

Figure S9. Junction area for droplet formation.

Impact of Tween 20 on CtHSD activity

To investigate whether Tween 20 has a direct negative impact on CtHSD activity, monophasic reactions were performed in 200 µL in a 96-well microtiter plate containing 20 mM Tris-HCl buffer, 25 mM NaCl, pH 9, 2 mM NAD^+, 2.5 µg/mL CtHSD, 1 mM testosterone (from a stock dissolved in 36% (w/v) 2-hydroxypropyl-β-cyclodextrin in H_2O), and varying concentration of Tween 20 (0, 0.5, 1, 2, 5 and 8 %). Each reaction was followed spectrophotometrically by measuring the absorbance of formed NADH at 340 nm and room temperature. Resulting enzyme activities were calculated from the respective absorbance increase according to the law of Lambert-Beer using a molar extinction coefficient ε for NADH of 6300 M^{-1} cm^{-1}.

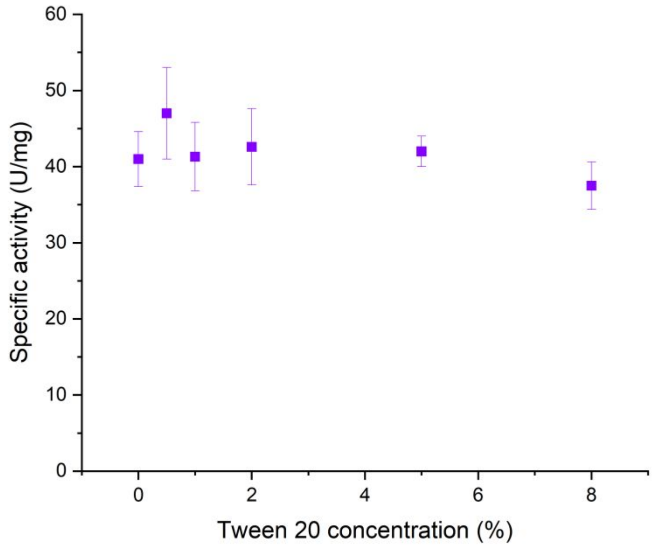

Figure S10. Specific activity of CtHSD at increasing Tween 20 concentration in 20 mM Tris-HCl buffer, 25 mM NaCl, pH 9 at room temperature. Error bars indicate standard deviations of triplicate measurements.

Residual enzyme activity determination after the two-phase reactions

To investigate the impact of the organic phase on enzyme inactivation after reactions on chip, monophasic reactions were performed in 200 µL in a 96-well microtiter plate containing 20 mM Tris-HCl buffer, 25 mM NaCl, pH 9, 2 mM NAD^+, 1.35 µg/mL CtHSD (taken from the aqueous phase of the reaction mixture after two-phase reaction for 2 min), and 1 mM testosterone (from a stock dissolved in 36% (w/v) 2-hydroxypropyl-β-cyclodextrin in H_2O). Each reaction was followed spectrophotometrically by measuring the absorbance of formed NADH at 340 nm and room temperature. Resulting enzyme activities were calculated from the respective absorbance increase according to the law of Lambert-Beer using a molar extinction coefficient ε for NADH of 6300 M^{-1} cm^{-1}, and compared to the respective enzyme activity of CtHSD before the two-phase reaction to obtain residual activities.

Table S2. Residual enzyme activity determination after two-phase reactions for 2 min.

Two-phase reaction	Specific enzyme activity [U/mg] before the two-phase reaction	Specific enzyme activity [U/mg] after the two-phase reaction	Residual activity [%]
Microfluidics	52.9 ± 5.2	52.7 ± 3.5	100

Determination of the testosterone partition coefficient

In a 2 mL vial, 750 µL 20 mM Tris-HCl, 25 mM NaCl, pH 9.0 and 250 µL TBME containing different concentrations of testosterone (10, 20 and 40 mM, resulting in final testosterone concentrations of 2.5, 5 and 10 mM in 1 mL volume) were combined and shaken at 1.100 rpm in a ThermoMixer C (Eppendorf) for 1 h at 25˚C. Afterwards, shaking was stopped and reactions were standing for 20 min for phase separation. The aqueous phase was removed and analyzed via HPLC to determine the testosterone concentration in the aqueous phase based on a respective calibration curve. The resulting partition coefficient Ptestosterone (**Table S3**) was calculated according to equation (**Equation S6**)

$P_{testosterone} = \frac{c_{TBME}}{c_{water}}$ (**Equation S6**)

where c_{TBME} refers to the testosterone concentration in the organic (TBME) phase and c_{water} to the testosterone concentration in the aqueous phase.

Table S3. Determination of the testosterone concentration in the aqueous phase and the resulting partition coefficient of testosterone $P_{testosterone}$ in a two-phase system consisting of 25 % TBME and 75 % aqueous phase (20 mM Tris-HCl, 25 mM NaCl, pH 9.0). For each testosterone concentration the experiment was performed in quintuplicate. For calculation of $P_{testosterone}$, the testosterone concentration in the organic phase was approximated to correspond to the applied testosterone concentration in the TBME phase.

Testosterone concentration in 1 mL total volume [mM]	Testosterone concentration in the applied organic (TBME) phase [mM]	Measured testosterone concentration in the aqueous phase [µM]	Ptestosterone
2.5	10	47.2 ± 2.6	212
5	20	96.8 ± 4.6	206
10	40	192.5 ± 40.4	208

The molecular model of testosterone

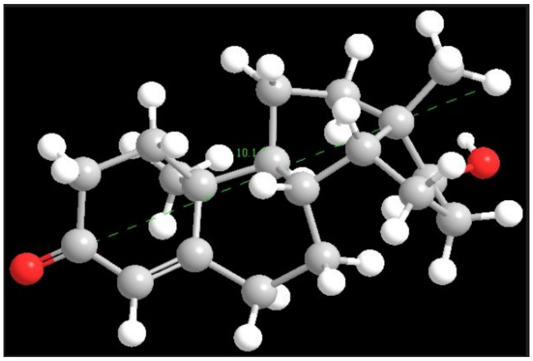

Figure S11. Molecular model of testosterone (made by ChemDraw 3D) showing maximum molecule diameter (10.1 Å).

Reference

(1) Eilert, L.; Schallmey, A.; Kaspar, F. UV-Spectroscopic Detection of (Pyro-)Phosphate with the PUB Module. *Anal Chem* **2022**, *94* (8), 3432–3435. https://doi.org/10.1021/acs.analchem.1c05356.

(2) Kaspar, F.; Seeger, M.; Westarp, S.; Köllmann, C.; Lehmann, A. P.; Pausch, P.; Kemper, S.; Neubauer, P.; Bange, G.; Schallmey, A.; Werz, D. B.; Kurreck, A. Diversification of 4′-Methylated Nucleosides by Nucleoside Phosphorylases. *ACS Catal* **2021**, *11* (17), 10830–10835. https://doi.org/10.1021/acscatal.1c02589.

(3) Xiang, L.; Kaspar, F.; Schallmey, A.; Constantinou, I. Two-Phase Biocatalysis in Microfluidic Droplets. *Biosensors (Basel)* **2021**, *11* (11), 407. https://doi.org/10.3390/bios11110407.

(4) Čech, J.; Přibyl, M.; Šnita, D. Three-Phase Slug Flow in Microchips Can Provide Beneficial Reaction Conditions for Enzyme Liquid-Liquid Reactions. *Biomicrofluidics* **2013**, *7* (5). https://doi.org/10.1063/1.4821168.

(5) Hernández, A.; Cano, M. P. High-Pressure and Temperature Effects on Enzyme Inactivation in Tomato Puree. *J Agric Food Chem* **1998**, *46* (1), 266–270. https://doi.org/10.1021/jf970455g.

(6) Islam, Md. N.; Zhang, M.; Adhikari, B. The Inactivation of Enzymes by Ultrasound—A Review of Potential Mechanisms. *Food Rev. Int.* **2014**, *30* (1), 1–21. https://doi.org/10.1080/87559129.2013.853772.

(7) Zumdahl, S. L. M. Lab Manual. In *Cengage Learning*; 2013; pp ix–xv.

(8) Bach, H.; Neuroth, N. The Properties of Optical Glass. *Springer* **2012**, 1–11.

(9) Erfle, P.; Riewe, J.; Bunjes, H.; Dietzel, A. Stabilized Production of Lipid Nanoparticles of Tunable Size in Taylor Flow Glass Devices with High-Surface-Quality 3D Microchannels. *Micromachines (Basel)* **2019**, *10* (4), 220. https://doi.org/10.3390/mi10040220.

(10) *https://imagej.net/plugins/denoiseg.*

(11) Buchholz, T.-O.; Prakash, M.; Krull, A.; Jug, F. DenoiSeg: Joint Denoising and Segmentation. **2020**.

(12) Ershov, D.; Phan, M.-S.; Pylvänäinen, J. W.; Rigaud, S. U.; Le Blanc, L.; Charles-Orszag, A.; Conway, J. R. W.; Laine, R. F.; Roy, N. H.; Bonazzi, D.; Duménil, G.; Jacquemet, G.; Tinevez, J.-Y. TrackMate 7: Integrating State-of-the-Art Segmentation Algorithms into Tracking Pipelines. *Nat Methods* **2022**, *19* (7), 829–832. https://doi.org/10.1038/s41592-022-01507-1.

(13) Stringer, C.; Wang, T.; Michaelos, M.; Pachitariu, M. Cellpose: A Generalist Algorithm for Cellular Segmentation. *Nat Methods* **2021**, *18* (1), 100–106. https://doi.org/10.1038/s41592-020-01018-x.

(14) Legland, D.; Arganda-Carreras, I.; Andrey, P. MorphoLibJ: Integrated Library and Plugins for Mathematical Morphology with ImageJ. *Bioinformatics* **2016**, *32* (22), 3532–3534. https://doi.org/10.1093/bioinformatics/btw413.
